# Supplementary material for: RECITAL: a non-inferiority randomised control trial evaluating a virtual fracture clinic compared with in-person care for people with simple fractures (study protocol)
Source: BMJ Open. 2024 Feb 5;14(2):e080800. doi: 10.1136/bmjopen-2023-080800 (PMC10859974; doi:10.1136/bmjopen-2023-080800)
Supplement: Supplementary data [file bmjopen-2023-080800supp001.pdf]

PARTICIPANT INFORMATION SHEET

Do patients with simple fractures have similar functional outcomes when comparing a virtual against an in-person fracture clinic?

**Title**	Effects of virtual fracture clinic care compared with in-person fracture clinic care on physical function in people with simple fractures: a non-inferiority randomised trial.
**Short Title**	fractu**RE** **C**lin**I**c **T**ri**AL** (RECITAL)
**Protocol Number**	2023/ETH01038
**Project Sponsor**	RPA Virtual Hospital
**Coordinating Principal Investigator**	• Dr Adrian Traeger, Research Fellow, Institute for Musculoskeletal Health, The University of Sydney.
**Associate Investigator(s)**	• Mr Min Jiat Teng, Physiotherapist, RPA Virtual Hospital; Chief Investigator & PhD Candidate, Institute for Musculoskeletal Health.<br>• Ms Miranda Shaw, General Manager, RPA Virtual Hospital, SLHD.<br>• Dr Owen Hutchings, Clinical Director, RPA Virtual Hospital, SLHD.<br>• A/Prof Mark Horsley, Deputy Director Neurosciences, Bone & Joint, SLHD.<br>• Dr Jeffrey Petchell, Head of Orthopaedic Department & Director of Trauma, Royal Prince Alfred Hospital, SLHD.<br>• Prof Chris Maher, Director, Institute for Musculoskeletal Health, The University of Sydney.<br>• Dr Tessa Copp, Postdoctoral Research Fellow, Sydney Health Literacy Lab, The University of Sydney.<br>• Dr Kristen Pickles, Postdoctoral Research Fellow, Sydney Health Literacy Lab, The University of Sydney.<br>• Ms Rong Liu, Research Officer (Health Economics), RPA Virtual Hospital, SLHD.<br>• Ms Alison Drayton, Consumer Representative.<br>• Ms Isabella Khoudair, Physiotherapist, RPA Virtual Hospital, SLHD.<br>• Mr Ben Warnock, Physiotherapist, RPA Virtual Hospital, SLHD.
**Location**	• RPA Virtual Hospital<br>• RPA Fracture Clinic

Teng MJ, *et al. BMJ Open* 2024; 14:e080800. doi: 10.1136/bmjopen-2023-080800

1. Introduction

You are invited to take part in a research study that will compare two existing models of care for managing patients with simple fractures. The aim of the study is to understand whether virtual clinics are comparable to in-person clinics in terms of function, recovery and other outcomes.

The study is being conducted within Sydney Local Health District (SLHD) by Min Jiat Teng (Physiotherapist, SLHD) as part of the requirements for a Doctor of Philosophy degree under the supervision of Dr Adrian Traeger (Senior Research Fellow, Institute for Musculoskeletal Health).

This Participant Information Sheet (PIS) will tell you what is involved in the study and help you decide whether or not you wish to take part. Please read this information carefully. If there is anything you do not understand or would like further information about, please ask Min Jiat Teng on 0460 001 381 or min.teng@health.nsw.gov.au. Before you make a decision, please feel free to talk things over with a relative, a friend or your doctor.

2. Study Procedures

Your condition has been discussed with the orthopaedic doctors at RPA to ensure it can be managed at either the virtual clinic or in-person clinic.

If you agree to participate in this study, you will be asked to sign an e-consent form online via a link provided by the study team.

Once you give informed consent, you will be randomly assigned to receive your care with RPA in-person fracture clinic at Camperdown or via the virtual fracture clinic.

If you are assigned to our virtual clinic, you will receive care from a physiotherapist via video calls. There will be 3 scheduled appointments that take approximately 30 minutes each, and your follow-ups will usually be at 2 and 6 weeks after your injury. You will not need to travel to hospital for your appointments.

If assigned to our traditional in-person clinic, you will attend appointments here at the hospital on Missenden Road, Camperdown. You will be provided a check-in time with the clinic, and the staff will assess you and provide a follow-up plan based on the assessments.

We do not know if one of these two ways of following people up is better than the other.

You will receive a link via email or SMS to complete an online survey at 3 different time-points – when enrolled into the study, 6 weeks after that, and again in another 6 weeks. Each survey will take less than 5 minutes to complete. The surveys will ask about your experiences with the care you have received and aspects of your recovery. You will receive an email or SMS 2 days prior to each milestone, reminding you to complete your surveys. You will receive two reminders via email or SMS, followed by a phone call by a SLHD staff if you do not complete your surveys.

Your clinical records will be maintained either on paper or electronically at SLHD as per current processes. The surveys you complete will be stored online in the secure ‘REDCap database’, which is managed by SLHD. Your clinical and research data on REDCap will be de-identified, and can only be re-identified through a data linkage process using a unique ID code.*

Your medical records may be accessed by the clinicians listed in this study if they are relevant to this research. This may include your paper and electronic medical records from the hospital,

and/or the radiology scans and reports conducted out of the hospital (e.g. if your xrays were conducted at a private radiology centre).

Data from this study may be published in peer-reviewed medical journals, however you will not be personally identifiable.

If the study data will be used for future research purposes and/or shared with national and international collaborators, Ethics Approval will be required prior to accessing any non-identifiable data.

*Data linkage is a method of bringing together information, from different sources, but relating to the same individual.

Qualitative interview sub-study

Patients in the virtual clinic may be contacted after discharge inviting you to participate in a phone or online interview to explore your experiences with the virtual fracture clinic. The interview will take 30 to 60 minutes, and will be audio/video recorded. If you do not want a video recording, you will be able to turn off your camera in the Zoom meeting. Audio from the interview recording will be transcribed and will not contain any details that will identify you.

The interview will be conducted by researchers from The University of Sydney or SLHD who are not part of your treating team. The interview will ask you about your experiences, feelings and expectations of the virtual fracture clinic.

3. Risks

All medical procedures - whether for diagnosis or treatment, routine or experimental – involve some level of risk. The table below displays some of these risks and the respective mitigation strategy.

Possible Risk/Side Effect	When may this occur?	Mitigation strategy
Telehealth privacy risk	Small risk that patient data or information is intercepted electronically during videoconsults or emails.	The study will only use programs that are approved by SLHD for clinical records, videocalls, exercise programs and data management.
Clinical risk	Small risk that a condition may be missed or mis-diagnosed as the clinician is unable to physically assess patients via video consult.	Only patients who meet the strict clinical criteria are considered for this study. All cases will be screened with the orthopaedic surgeon to ensure clinical suitability to be managed either in person or by video consult.

If you wish to talk to someone outside the research team due to any distress caused to you by this study, you can contact:

- Executive Officer – Clinical Trials on 02 9515 8200.
- Research and Evaluation Manager, RPA Virtual Hospital on 02 9515 0248.
- Beyond Blue on 1300 224 636.
- Mental Health Line on 1800 011 511.

4. Benefits

Teng MJ, *et al. BMJ Open* 2024; 14:e080800. doi: 10.1136/bmjopen-2023-080800

Your participation in this study will also further medical knowledge and may improve treatment of virtual care for patients with simple injuries in the future.

5. Compensation for injuries or complications

If you suffer any injuries or complications as a result of this study, you should contact the study clinician as soon as possible, who will assist you in arranging appropriate medical treatment. If you are eligible for Medicare, you can receive any medical treatment required to treat the injury or complication, free of charge, as a public patient in any Australian public hospital.

In addition, you may have a right to take legal action to obtain compensation for any injuries or complications resulting from the study. Compensation may be available if your injury or complication is sufficiently serious and is caused by the services you received as part of this study, or by the negligence of one of the parties involved in the study (for example, the researcher, the hospital, or the treating doctor). You do not give up any legal rights to compensation by participating in this study.

6. Costs

Aside from giving up your time, there are no costs of participating in this study. You will receive a $50 eGift Card (WISH GiftCard) after completing all the surveys (at recruitment, 6 weeks and 12 weeks) to thank you for your time. You will receive an additional $50 eGift Card if you participate in the interview.

7. Voluntary Participation

Participation in this study is entirely voluntary. You do not have to take part. If you do take part, you can withdraw at any time without having to give a reason by contacting the study coordinator, Min Jiat Teng on 0460 001 381. Whatever your decision, please be assured that it will not affect your medical treatment or your relationship with the staff who are caring for you.

If you decide to withdraw from the study, we will not collect any more study-related information from you, although information already collected will be retained to ensure that the results of the research project can be measured properly and to comply with law. You should be aware that data collected up to the time you withdraw will form part of the research project results. If you do not want your data to be included, you must tell the researchers when you withdraw from the project. It will not be included in the study results, unless we have already analysed and published the results in aggregate form (you will not be personally identifiable).

8. Confidentiality

All the information collected from you for the study will be strictly confidential and will be stored on a secure research database (SLHD's REDCap server). This web-based software is managed and supported by the Clinical Research Centre and the SLHD Digital Health and Innovation (DH&I) department. This server is stable and is backed up daily in compliance with national, state and district privacy and confidentiality obligations. Only the investigators named on this research project or your treating clinicians will have access to it.

The data will be analysed by the researchers at RPA Virtual Hospital and The University of Sydney. All data included in journal publications and presentations will be de-identified*. The files will be retained for 15 years from the day the study is completed, after which they will be securely destroyed.

Any personally identifiable data such as your name, date of birth and e-consent form will be kept strictly confidential, separate from your survey data within REDCap. The data can only

be linked using a unique ID code. Only the named investigators and the clinicians providing care will have access to the data. The **rpa**virtual General Manager listed on this Participant Information Sheet will be the data custodian for this research.

*de-identified data means that you/your information will not be identifiable

9. Storage of Data

The SLHD software licence for REDCap (Research Electronic Data Capture) will be used for to manage the collection and storage of research data. REDCap is a secure, web-based, non-commercial, data management tool designed for research purposes. Data collected by REDCap is stored on servers in the SLHD data centre. Data is secured and backed-up to maintain your privacy and confidentiality in line with national, state and district standards.

10. Future use of Data

The data collected in this project may also be used in future research studies. The results of this study and de-identified data may be shared in the future with national and international collaborators. Any stored data that is used for related or future research will first be reviewed and approved by an appropriately constituted Ethics Committee.

11. Conflicts of Interest

The following investigators (AT, MS, OH, CM, TC, KP, JZ, IA, RL, AD) have no conflict on interests to declare. The SLHD clinicians (MJT, IK, BW, MH, JP) may deliver care to participants in either study groups as part of their usual clinical role provided at a public hospital. The SLHD clinicians will receive no financial or non-financial benefits for conducting this research, nor will RPA Virtual or RPA Hospitals receive any financial or other benefits.

Min Jiat Teng will be conducting this study in partial fulfillment of the requirements of a Doctor of Philosophy (Medicine and Health) degree under the supervision of Dr Adrian Traeger, Prof Christopher Maher, Dr Tessa Copp and Dr Kristen Pickles. This study has received funding from Sydney Research and the NHMRC 2022 MRFF Clinician Researchers - Nurses Midwives and Allied Health grant.

12. Further Information

When you have read this information, Min Jiat Teng will discuss it with you further and answer any questions you may have. If you would like to know more at any stage, please feel free to contact him on 0460 001 381 or min.teng@health.nsw.gov.au.

This information sheet is for you to keep.

13. Ethics Approval and Complaints

This study has been approved by the Ethics Review Committee (RPAH Zone) of the Sydney Local Health District. Any person with concerns or complaints about the conduct of this study should contact the Executive Officer on 02 9515 6766 and quote protocol number 2023/ETH01038.

Effects of virtual fracture clinic care compared with in-person fracture clinic care on physical function in people with simple fractures: a non-inferiority randomised trial.

Do patients with simple fractures have similar functional outcomes when comparing a virtual against an in-person fracture clinic?

PARTICIPANT CONSENT FORM

I, ____ *[full name]*

of ____ *[address]*

- I have read and understood the Participant Information Sheet on the above-named research study and have had the opportunity to discuss the study with the research team if required.
- I have been made aware of the procedures involved in the study, including any known or expected inconvenience, risk, discomfort or potential side effect and of their implications as far as they are currently known by the researchers.
- I understand that the interview discussion will be audio and/or video-recorded, and will then be transcribed and be kept in a manner in which I cannot be identified for analysis.
- I understand that my participation in this study will allow my clinicians and the study coordinator to have access to my medical record, as described in the Participant Information Sheet.
- I understand that my de-identified data may be used for future research.
- I would like to receive a copy of the study results when they become available.

  ❑ Yes ❑ No
- I understand that, during the course of this study, my medical records may be accessed by the research staff at RPA Virtual Hospital, by regulatory authorities or by the Ethics Committee approving the research in order to verify results and determine that the study is being carried out correctly.
- I understand that the SLHD software license for REDCap (Research Electronic Data Capture) will be used to manage the collection and storage of my research data.
- I have had an opportunity to ask questions and I am satisfied with the answers I have received.
- I freely choose to participate in this study and understand that I can withdraw at any time.
- I consent to the future use of any data I provide for research purposes. I understand that before they can use any data I provide, they must seek additional ethics approval.
- I understand that my participation and data will be kept strictly confidential and secure.
- I hereby agree to participate in this research study.
- I consent to the storage and use of my information collected from me for use, as described in the relevant section of the Participant Information Sheet, for:
  - This specific research project

Teng MJ, *et al. BMJ Open* 2024; 14:e080800. doi: 10.1136/bmjopen-2023-080800

- Other research that is closely related to this research project

My email address is ____

(a link to the survey will be send to this address)

Participant Name: ____

Participant Signature: ____

Date: ____

Teng MJ, *et al. BMJ Open* 2024; 14:e080800. doi: 10.1136/bmjopen-2023-080800
