# Supplementary material for: RECITAL: a non-inferiority randomised control trial evaluating a virtual fracture clinic compared with in-person care for people with simple fractures (study protocol)
Source: BMJ Open. 2024 Feb 5;14(2):e080800. doi: 10.1136/bmjopen-2023-080800 (PMC10859974; doi:10.1136/bmjopen-2023-080800)
Supplement: Author's manuscript [file bmjopen-2023-080800.draft_revisions.pdf]

BMJ Open

BMJ Open is committed to open peer review. As part of this commitment we make the peer review history of every article we publish publicly available.

When an article is published we post the peer reviewers' comments and the authors' responses online. We also post the versions of the paper that were used during peer review. These are the versions that the peer review comments apply to.

The versions of the paper that follow are the versions that were submitted during the peer review process. They are not the versions of record or the final published versions. They should not be cited or distributed as the published version of this manuscript.

BMJ Open is an open access journal and the full, final, typeset and author-corrected version of record of the manuscript is available on our site with no access controls, subscription charges or pay-per-view fees (http://bmjopen.bmj.com).

If you have any questions on BMJ Open's open peer review process please email info.bmjopen@bmj.com

BMJ Open

RECITAL: A non-inferiority randomised control trial evaluating a virtual fracture clinic compared with in-person care for people with simple fractures

Journal:	*BMJ Open*
Manuscript ID	bmjopen-2023-080800
Article Type:	Protocol
Date Submitted by the Author:	11-Oct-2023
Complete List of Authors:	Teng, Min Jiat; The University of Sydney Faculty of Medicine and Health, Institute for Musculoskeletal Health, Sydney School of Public Health; Sydney Local Health District, RPA Virtual Hospital<br>Zadro, Joshua; The University of Sydney Faculty of Medicine and Health, Institute for Musculoskeletal Health, Sydney School of Public Health<br>Pickles, Kristen; The University of Sydney Faculty of Medicine and Health, Sydney Health Literacy Lab, Sydney School of Public Health<br>Copp, Tessa; The University of Sydney Faculty of Medicine and Health, Sydney Health Literacy Lab, Sydney School of Public Health<br>Shaw, Miranda; Sydney Local Health District, RPA Virtual Hospital<br>Khoudair, Isabella; Sydney Local Health District, RPA Virtual Hospital<br>Horsley, Mark; Sydney Local Health District, Department of Orthopaedic Surgery, Royal Prince Alfred Hospital<br>Warnock, Benjamin; Sydney Local Health District, RPA Virtual Hospital<br>Hutchings, Owen; Sydney Local Health District, RPA Virtual Hospital<br>Petchell, Jeffrey; Sydney Local Health District, Department of Orthopaedic Surgery, Royal Prince Alfred Hospital<br>Ackerman, Ilana; Monash University, School of Public Health and Preventive Medicine<br>Drayton, Alison; Consumer representative<br>Liu, Rong; Sydney Local Health District, RPA Virtual Hospital; The George Institute for Global Health, University of New South Wales<br>Maher, Christopher; The University of Sydney Faculty of Medicine and Health, Institute for Musculoskeletal Health, Sydney School of Public Health<br>Traeger, Adrian; The University of Sydney Faculty of Medicine and Health, Institute for Musculoskeletal Health, Sydney School of Public Health
Keywords:	Telemedicine < BIOTECHNOLOGY & BIOINFORMATICS, ORTHOPAEDIC & TRAUMA SURGERY, Randomized Controlled Trial, Protocols & guidelines < HEALTH SERVICES ADMINISTRATION & MANAGEMENT, Patient Reported Outcome Measures

SCHOLARONE™
Manuscripts

RECITAL: A non-inferiority randomised control trial evaluating a virtual fracture clinic compared with in-person care for people with simple fractures

Min Jiat Teng[1,3], Joshua R Zadro[1], Kristen Pickles[2], Tessa Copp[2], Miranda Shaw[3], Isabella Khoudair[3], Mark Horsley[4], Benjamin Warnock[3], Owen Hutchings[3], Jeffrey Petchell[4], Ilana Ackerman[5], Alison Drayton[6], Rong Liu[3,7], Chris G Maher[1], and Adrian C Traeger[1]

[1]The Institute for Musculoskeletal Health, The University of Sydney and Sydney Local Health District, Gadigal Country, Sydney, New South Wales, Australia

[2]University of Sydney, Sydney Health Literacy Lab, School of Public Health, Faculty of Medicine and Health, Sydney, New South Wales, Australia

[3]RPA Virtual Hospital, Sydney Local Health District, Sydney, New South Wales, Australia

[4]Department of Orthopaedic Surgery, Royal Prince Alfred Hospital, Sydney Local Health District, Sydney, New South Wales, Australia

[5]School of Public Health and Preventative Medicine, Monash University, Melbourne, Victoria, Australia

[6]Consumer representative

[7]The George Institute for Global Health, University of New South Wales, Sydney, New South Wales, Australia

CORRESPONDENCE

Min Jiat Teng
Min.Teng@health.nsw.gov.au
The Institute for Musculoskeletal Health
Level 10N, King George V Building, Royal Prince Alfred Hospital
PO Box M179, Missenden Road Camperdown, NSW, AUS 2050

For peer review only - http://bmjopen.bmj.com/site/about/guidelines.xhtml

KEYWORDS

Telemedicine, orthopaedic, randomised controlled trial, protocol, Patient-Specific Functional Scale

WORD COUNT: 3642 words

For peer review only

For peer review only - http://bmjopen.bmj.com/site/about/guidelines.xhtml

ABSTRACT

INTRODUCTION

Most simple undisplaced fractures can be managed without surgery by immobilising the limb with a splint, prescribing medication for pain, and providing advice and early rehabilitation. Recent systematic reviews based on retrospective observational studies have reported that virtual fracture clinics can deliver follow-up care that is safe and cost-effective. However, no randomised controlled trial has investigated if a virtual fracture clinic can provide non-inferior physical function outcomes compared to an in-person clinic for patients with simple fractures.

METHODS AND ANALYSIS

312 participants will be recruited from two metropolitan hospitals located in Sydney, Australia. Adult patients will be eligible if they have an acute simple fracture that can be managed with a removable splint, and is deemed appropriate for follow-up at either the virtual or in-person fracture clinic by an orthopaedic doctor. Patients will not be eligible if they have a complex fracture that requires a cast or surgery. Eligible participants will be randomised to receive their follow-up care either at the virtual or the in-person fracture clinic. Participants at the virtual fracture clinic will be reviewed within five days of receiving a referral through video calls with a physiotherapist. Participants at the in-person fracture clinic will be reviewed within seven to ten days of receiving a referral with an orthopaedic doctor. The primary outcome will be the patient's function measured using the Patient-Specific Functional Scale at 12 weeks. Secondary outcomes will include health-related quality of life, patient-reported experiences, pain, health cost, healthcare utilisation, medication use, and adverse events.

ETHICS AND DISSEMINATION

The study has been approved by the Sydney Local Health District Ethics Review Committee (RPAH Zone) (X23-0200). The trial results will be submitted for publication in a reputable international journal and will be presented at professional conferences.

TRIAL REGISTRATION NUMBER

https://www.anzctr.org.au/Trial/Registration/TrialReview.aspx?ACTRN=12623000934640; ACTRN12623000934640

For peer review only - http://bmjopen.bmj.com/site/about/guidelines.xhtml

STRENGTH AND LIMITATIONS OF THIS STUDY

- Pragmatic clinical trial embedded within two existing fracture clinics at two urban hospitals
- Measures hospital-level outcomes as well as patient outcomes and experiences
- Blinding of therapist or participants is not possible, although participants are blinded to the study hypothesis
- Methods and results from this trial may inform the evaluation of other virtual musculoskeletal services

For peer review only

For peer review only - http://bmjopen.bmj.com/site/about/guidelines.xhtml

INTRODUCTION

In 2019, there were 178 million new fractures reported globally, an increase of 33.4% since 1990.[1] In Australia, the treatment costs of osteoporosis-related fractures were estimated to be A$2.34 billion in 2017.[2] With increasing numbers of people requiring care for their fractures, the burden on outpatient fracture clinics has also increased, causing long clinic wait times, and productivity losses for patients and carers from missing school or work.[3,4] The recent pandemic further strengthens the requirement for health system efficiency.

Most simple fractures, including minimally displaced fractures, can be managed conservatively without surgery. These stable fractures are managed with short-term immobilisation, advice, pain relieving medication, and early rehabilitation.[5] Traditional physical assessments at the outpatient clinic may not be required for conditions that have a clear prognosis and have been shown to recover well with conservative management.[6]

Published studies have shown that virtual fracture clinics can manage patients with simple fractures.[7] Patients receive advice and management through phone calls and written handouts, rather than attending the outpatient clinic in-person. Retrospective observational studies have reported that this virtual model is associated with good patient satisfaction, increased cost-efficiency for the hospital system, fewer adverse events and reduced presentations to in-person clinics.[8]

Despite a rise in virtual fracture clinics since the recent pandemic, robust evaluations of their safety, effectiveness and cost-effectiveness are lacking. A recent systematic review of 21 publications suggested that virtual fracture clinics could provide safe and cost-effective outpatient care to patients with acute fractures, though none of the included studies were randomised controlled trials.[8] It is currently unknown whether virtual care for patients with simple fractures leads to non-inferior outcomes compared with in-person care.

We have designed a clinical trial to evaluate the effectiveness of a virtual fracture clinic for patients with simple fractures. The primary outcome of this trial is to determine whether virtual care produces non-inferior physical function outcomes compared to in-person care for patients with simple fractures at 12-weeks follow-up, measured using the Patient-Specific Functional Scale (PSFS). The secondary outcomes include pain, quality of life, patient-reported

For peer review only - http://bmjopen.bmj.com/site/about/guidelines.xhtml

experience measures, cost-effectiveness, healthcare utilisation, medication use and safety. A qualitative sub-study will be conducted to explore the experiences, feelings and expectations of patients who use the virtual fracture clinic.

METHODS AND ANALYSIS

Design

The Fracture Clinic Trial (RECITAL) is a prospective two-arm, parallel group randomised controlled trial, using a non-inferiority design with nested economic and process evaluations. We chose a non-inferiority randomised controlled trial design as both study groups are existing hospital services, and the virtual clinic is expected to have outcomes that are at least no worse than the usual follow-up care at in-person fracture clinic. This trial has been prospectively registered with the Australian New Zealand Clinical Trials Registry (ACTRN12623000934640). This document describes the trial protocol according the Standard Protocol Items: Recommendations for Interventional Trials (SPIRIT) 2013 Statement.[9]

Setting

RECITAL will compare two existing models of care provided at two metropolitan public hospitals within Sydney Local Health District (SLHD) in New South Wales, Australia. The virtual fracture clinic (intervention group) is located at RPA Virtual Hospital (**rpa**virtual), while the in-person fracture clinic (control group) is situated at the Royal Prince Alfred (RPA) Hospital. **rpa**virtual is Australia's first virtual hospital established in February 2020 to enable patients to receive hospital-level care at home through virtual means (e.g. video calls or remote monitoring), rather than visiting the traditional hospital for their healthcare needs.[10]

Eligibility criteria

Patients referred to the virtual fracture clinic from RPA Hospital Emergency Department, local General Practices, and the (in-person) RPA Fracture Clinic will be identified and screened by a virtual fracture clinic physiotherapist and an orthopaedic doctor to determine if the patient is

For peer review only - http://bmjopen.bmj.com/site/about/guidelines.xhtml

suitable for either model of care (virtual or in-person). The RECITAL study staff will contact the eligible patients to invite them into this study. Figure 1 illustrates the trial design.

Patients will be invited to participate if they meet the following criteria:

- Have an acute (<6 weeks old) simple fracture, such as base of fifth metatarsal, ankle Weber A, Mason I radial head or clavicle, that can be managed using a removable orthoses (e.g. shoulder immobiliser, CAMboot or wrist splint).
- Aged ≥18 years.
- Has access to a phone.
- Is willing to participate and comply with the study requirements.

Patients will be excluded if they have:

- Complex or significantly displaced fracture, including pathological, open, unstable or spinal fractures requiring a cast or surgical management.
- Neurovascular concerns.
- A condition not managed by RPA Hospital Orthopaedics Department.
- Are unable to attend the in-person fracture clinic within the recommended follow-up time.

Patients who consent to participate and complete their baseline measures will be enrolled in this study. Informed consent and study data will be collected and managed using the Research Electronic Data Capture (REDCap) tool hosted at SLHD.[11,12] The randomisation schedule will be computer-generated using REDCap's randomisation model, and will be stratified in random blocks of 4, 6, 8 and 10 to ensure equal numbers in both groups and concealed allocation. A biostatistician not involved in this study will set up the allocation schedule and upload it into REDCap. Only the biostatistician will be aware of the allocation to ensure concealment. The study coordinator will randomise the patients to the study groups. Participants randomised to virtual care who agree to participate in the qualitative sub-study will be purposively selected for an interview according to their age, employment status, tertiary education level, type of injury and discharge status. Selected participants will be contacted once they are discharged from the clinic to ensure their complete experience with the virtual clinic is captured.

For peer review only - http://bmjopen.bmj.com/site/about/guidelines.xhtml

Interventions

Both study groups reflect current processes within existing clinics at the participating hospitals.

Virtual Fracture Clinic (VFC) (Intervention Group)

Patients randomised to the VFC (intervention) group will be contacted via phone and email to organise an initial follow-up with a physiotherapist; usually within 5 days after their referral is received. Patients are sent an email with their appointment details and a fracture management fact sheet. The fact sheet explains their clinical condition, expected recovery, early rehabilitation exercises, activity limitations, and information on care escalation. These fact sheets were adapted with permission from the Royal Melbourne Hospital's Virtual Fracture Clinic.

All patients are offered a video-consultation with a physiotherapist unless they choose to have their review via phone. During the virtual consults, the physiotherapist conducts an assessment, discusses the x-ray findings, and provides a management plan. The virtual consult sessions are usually approximately 30 minutes. An email summary of the consultation, and follow-up appointment details are sent to the patient after the consultation. A Physitrack link may also be included in this email. Physitrack or PhysiApp is an internet-based program that allows patients to view videos of their prescribed exercises. Patients are usually offered a follow-up virtual appointment at 2- and 6-weeks post-fracture, or based on clinical need. Patients can contact the physiotherapist out of session if they have any concerns during their care period. Most patients are discharged from the VFC at 6-weeks post-fracture if there are no concerns. Patients will be supported with an interpreter, Aboriginal Cultural Support Team or with loaned devices and data as required. We will monitor patient adherence by the number of consults attended; and the number of ad hoc patient contacts via phone or email with the clinic.

In-person RPA Fracture Clinic (Control Group)

Patients randomised to the in-person clinic (control) group will be contacted via phone or email to provide a follow-up appointment at the in-person fracture clinic. Appointments usually occur 7 to 10 days after the referral is received, based on the next clinic of the on-call orthopaedic doctor. Clinical management and subsequent follow-ups of the control group will be determined

For peer review only - http://bmjopen.bmj.com/site/about/guidelines.xhtml

by the orthopaedic doctors at the in-person fracture clinic. Clinical management can include a physical assessment by a doctor, radiology scan, advice and exercises. A physiotherapist may be involved in the patient's care. Patients in the control group may receive written instructions about their recovery and exercises as per current processes. The in-person consult sessions are usually approximately 20 minutes. Current practice suggests that patients may attend the in-person fracture clinic once or twice within 6-weeks post-fracture. We will monitor patient adherence by the number of consults attended.

Staff at both study groups will be trained on the trial protocol and be regularly supported by study investigators to ensure adherence to the study.

Outcomes

The primary outcome for this study will be the participant's physical function assessed using the PSFS at 12-weeks. This self-reported tool has shown to be sensitive to change in patients with musculoskeletal problems, including the types of fractures included in this study.[13-15] Participants list up to five functional tasks at baseline, and score each task at baseline, and at 12-weeks, based on their level of ability – 0 (unable to perform activity) to 10 (able to perform activity at the same level as before the injury). Scores for each activity will be summed and calculated as an average of the total possible score for the participant (determined by the number of identified activities). We will compare the PSFS average scores between groups at 12-weeks as our primary measure. Table 1 summarises the outcome measures for this study.

Secondary measures for this study will include:

- Health-related quality of life measure assessed using the EuroQol 5D 5L (EQ-5D-5L) at baseline and 12-weeks,[16]
- Patient-reported experience measure assessed using the Generic Short Patient Experiences Questionnaire (GS-PEQ) at 6-weeks,[17]
- Pain assessed using the 0-10 Numerical Rating Scale (NRS) at baseline, 6- and 12-weeks,[18]

For peer review only - http://bmjopen.bmj.com/site/about/guidelines.xhtml

- Cost borne by the healthcare service, measured at 12-weeks. We will collect data from the electronic medical records (eMR), Sydney Local Health District Targeted Activity and Reporting System (STARS App) Dashboard, and the hospital's performance, data and finance departments to obtain the healthcare appointment duration, healthcare provider's hourly rate, any health services utilisation and corresponding cost (including but not limited to outpatient, inpatient, emergency department, pharmacy, radiology, pathology and primary care), any infrastructure setup and maintenance cost, managerial and administrative overhead.
- Cost borne by patients, measured through the patient survey designed specifically for this study at 12-weeks.
- Healthcare utilisation assessed using a survey designed specifically for this study at 12-weeks. The survey will collect the number of other healthcare appointments for management of their injury. We will also ascertain if the patients utilised any other healthcare services through the patient's eMR.
- Medication use assessed using a survey designed specifically for this study to assess the name and dose of prescription or over the counter medication for their injury at 6-weeks, and
- Adverse Events and Serious Adverse Events assessed using a survey designed specifically for this study at 6- and 12-weeks. We will also collect data from safety reports within the eMR, and the NSW Health Incident Management System (IMS+).

For peer review only

Sample size

A total sample of 312 participants will provide 90% power to detect a non-inferiority margin of 0.7 points on the 11-point PSFS with a 10% loss to follow-up, a standard deviation of 2.0, α of 5%, and a correlation score between baseline and final scores of 0.5 at 12-weeks.[19] A negative between-group difference of ≤0.7 points will indicate that the virtual fracture clinic is non-inferior to the in-person fracture clinic.

For peer review only - http://bmjopen.bmj.com/site/about/guidelines.xhtml

We chose a standard deviation of 2.0 as this is between the mean value of the standard deviation for the PSFS at follow-up in published studies that range from 1.7[20], 2.1[21] and 2.2[13]. The minimal important difference (MID) for the PSFS ranges from 1.3 (small change) to 2.7 (large change).[21,22] Guidelines suggest using a non-inferiority margin of 50% (or less preferably) of the treatment effect of standard care vs placebo.[23] Thus, we chose a between-group non-inferiority margin of 0.7 (50% of the MID of 1.3).

Blinding

The participants, therapists and assessors will not be blinded. The surveys administered during this trial are self-assessments completed by patients directly in REDCap who will be blinded to the study hypothesis. If required, an independent blinded assessor may contact the patient to assist them with completing their surveys.

Data collection methods

Patients will receive a unique link via email or phone message to complete all their surveys directly in REDCap. Patients will receive an email or phone message 2 days prior to each milestone, reminding them to complete their respective surveys. Two reminders followed by a phone call will be provided to patients who do not complete their surveys by the respective milestone. The treating clinicians may remind the participants to complete their surveys during their routine clinical reviews. The clinicians will not be able to complete, nor alter the results from the surveys. If requested, paper copies of the surveys may be sent to participants with their responses transcribed verbatim into REDCap by an independent blinded assessor not involved in this study.

Data management

All study data will be collected, logged and stored within SLHD's REDCap server. REDCap functions such as adding a field note (a brief descriptor of the question or answer), auto-calculations, and using data validation functions will be used to ensure data quality. The 'required' field is also used to ensure participants complete the mandatory questions prior to

For peer review only - http://bmjopen.bmj.com/site/about/guidelines.xhtml

submitting the survey. The questionnaires will be tested by clinicians and patients prior to implementation. The research team will have access through a personal login and password.

Statistical methods

An intention-to-treat analysis will be implemented after the database is cleaned and locked. Separate analyses will be conducted on each outcome. Descriptive statistics will be used for patient demographics and clinical characteristics. Categorical variables will be described with frequencies (%), and continuous variables will be described with means and standard deviations. Data will be analysed using STATA version 14 statistical software (StataCorp, College Station, TX) or R version 4.2.1 (R Foundation for Statistical Computing, Vienna, Austria. https://www.R-project.org/).

Primary analysis

Non-inferiority trials assess whether an intervention outcome is not clinically worse than a control. The PSFS score at 12-weeks post-randomisation is the primary outcome in this study and we have prospectively defined a non-inferiority margin (ΔT) of -0.7 points, which is the maximum difference we are prepared to tolerate and still consider virtual care not to be clinically inferior to in-person care. The null hypothesis is therefore that a difference of greater than ΔT exists in favour of in-person care (H0: $\Delta \leq -\Delta T$). This will be assessed by creating a 95% confidence interval, which should be entirely above the non-inferiority margin for the intervention to be declared non-inferior. The PSFS score will be compared between treatment groups as the dependent variable in a generalised linear regression model for the primary analysis adjusting for baseline PSFS variables. The treatment difference will be based on the estimate of adjusted means and 95% confidence intervals.

Secondary analysis

Secondary clinical outcomes will be analysed using logistic regression for binary outcomes and linear regression for continuous outcomes. Results from the analyses will be presented as point estimates with 95% confidence intervals. Baseline scores will be included in the model to

For peer review only - http://bmjopen.bmj.com/site/about/guidelines.xhtml

increase statistical precision. If more than 5% of data are missing, then imputation techniques may be considered.

Cost-effectiveness analysis

The economic evaluation will estimate the difference in the cost of resource inputs used by participants in the two arms of the trial, allowing comparisons to be made between the two models of care.

We will conduct a cost-effectiveness analysis to estimate the incremental-effectiveness ratios (ICERs) defined as: [cost of the virtual care - cost of in-person practice]/[effectiveness of the virtual care - effectiveness of in-person practice]. The effectiveness outcomes include PSFS, ED visit, rehospitalisation, and quality-adjusted life-years (QALYs). Costs for resource inputs will largely be derived from available local and national sources and estimated in line with best practice. Primary research using established accounting methods may also be required to estimate unit costs. Costs will be standardised to current prices where possible. The EQ-5D-5L outcomes will be used to generate QALYs, and the responses will be compared to the national Australian value set for the EQ-5D-5L.[16] Multiple imputation methods will be used to impute missing data and avoid biases associated with complete case analysis.

To estimate the uncertainty of ICERs, bootstrapping will be used to resample corresponding costs and effectiveness that will be observed in RECITAL, and the distribution of ICERs calculated from all resamples will be plotted on a cost-effectiveness plane. Subgroup analysis will be carried out to assess the equity impact of the interventions. One-way sensitivity analysis will be conducted around key cost variables. A cost-effectiveness acceptability curve (CEAC) will be plotted, which will provide information about the probability that an intervention is cost-effective, given the level of a decision maker's willingness to pay for each additional effectiveness outcome gain. The economic assessment method will adhere to the Consolidated Health Economic Evaluation Reporting Standards 2022 (CHEERS 2022).[24,25]

Qualitative interview analysis

The thematic analysis will be based on Braun and Clarke's six-phase framework.[26] After the interview recordings are transcribed verbatim, the research team will independently annotate

For peer review only - http://bmjopen.bmj.com/site/about/guidelines.xhtml

the transcripts to generate initial ideas and relevant phrases. A qualitative data analytic software (Nvivo) will be used to code and organise the data into themes. The topic guide may be modified between interviews to enable new emerging themes from the interviews to be explored more in depth with subsequent patients. The data will be reported according to the Consolidated Criteria for Reporting Qualitative Research (COREQ).

Data monitoring

Given the relatively low-risk nature of the intervention, a data safety and monitoring board will not be utilised in this study. The study coordinator will provide feedback (at least once per year) to the investigator team, which consists of orthopaedic doctors, senior researchers, hospital executives and a consumer.

Harms

Adverse and serious adverse events as defined by the National Health and Medical Research Council (NHMRC) will be monitored throughout this study.[27] Potential adverse events arising from this study include mis-diagnoses or missed diagnoses; emergency department re-presentations or surgical management of the fracture. All serious adverse events will be reported immediately to the investigator team and Human Research Ethics Committee.

Auditing

There are no planned audits for this study.

Consent or assent

The RECITAL study staff will contact all eligible patients to inform them about their follow-up options for their simple fractures using a standardised recruitment script. All patients who agree to have their follow-up care at one of the fracture clinic (virtual or in-person) will be invited to participate in this study. Study staff will send the patient a REDCap link via email or phone message for participants to view the study outline and requirements online, including the opportunity to download the Participant Information Sheet. If the patient agrees to participate, they will complete an e-Consent form within REDCap. Participants who choose not to

For peer review only - http://bmjopen.bmj.com/site/about/guidelines.xhtml

participate in the RECITAL study will be able to choose their follow-up at the virtual or in-person fracture clinic. Only patients who have completed the e-Consent form will be enrolled into this study.

Access to data

Only clinicians providing care to the participants and the study coordinator will have access to the identifiable data. All other investigators of this study will have access to the de-identified data. As per NHMRC requirements, the research data from this study will be retained for 15 years from the end of the trial.[28] Study protocol will be made available upon reasonable request.

Ancillary and post-trial care

Study participants are free to engage with other treatment providers such as their general practitioner or outpatient physiotherapist during and after this study for the management of their injury. These costs will not be borne by the study. This study will capture these visits to other health care providers for the management of this injury through the healthcare utilisation survey.

Dissemination policy

The trial results will be submitted for publication in reputable international journals and will be presented at relevant professional conferences. The results will also be disseminated to the media. Authorship eligibility will align with the International Committee of Medical Journal Editors.

Consumer involvement

AD is a co-investigator of this trial and had prior experiences at both fracture clinics investigated by this study. Facts sheets used by the VFC have been approved by the **rpa**virtual Consumer Group. This study will investigate the experiences of participants through the Generic Short Patient Experiences Questionnaire and qualitative interviews.

For peer review only - http://bmjopen.bmj.com/site/about/guidelines.xhtml

CONCLUSION

This trial has been designed to be embedded in usual clinical practice to evaluate two existing models of care at two urban public hospitals. Results from this trial will inform patients, clinicians, hospitals, policy makers and health funders globally about the effectiveness of a virtual fracture clinic.

ETHICS AND DISSEMINATION

Study has been approved by the Sydney Local Health District Ethics Review Committee (RPAH Zone) (X23-0200 & 2023/ETH01038; 30 June 2023). Any amendments to the trial protocol will require approval from the trial's Steering Committee and the ethics committee prior to implementation. Recruitment will commence in October 2023 and is expected to complete by September 2026.

AUTHOR CONTRIBUTIONS

ACT, CGM and MJT conceived the idea for the trial. All authors contributed to the trial design. MJT drafted the manuscript. All authors contributed intellectual content to the manuscript and approved the final version.

FUNDING STATEMENT

This work is supported by the National Health and Medical Research Council (NHMRC) 2022 Medical Research Future Fund (MRFF) Clinician Researchers – Nurses Midwives and Allied Health Grant Number 2022985 and Sydney Research 2022 Clinician Researcher Scholarship. The funding sources did not contribute to the trial design and will not have a role in the trial conduct, data analysis, interpretation, writing or reporting.

For peer review only - http://bmjopen.bmj.com/site/about/guidelines.xhtml

For peer review only

COMPETING INTERESTS STATEMENT

The following investigators (ACT, MS, OH, CGM, TC, KP, JZ, IA, RL, AD) have no conflict on interests to declare. The SLHD clinicians (MJT, IK, BW, MH, JP) may deliver care to participants in either study groups as part of their usual clinical role provided at the public hospitals. MJT will be conducting this study in partial fulfillment of the requirements of a Doctor of Philosophy (Medicine and Health) degree under the supervision AT, CGM, TC, KP and JZ.

REFERENCES

1. Wu A-M, Bisignano C, James SL, et al. Global, regional, and national burden of bone fractures in 204 countries and territories, 1990–2019: a systematic analysis from the Global Burden of Disease Study 2019. *The Lancet Healthy Longevity*. 2021;2(9):e580-e592. doi:10.1016/s2666-7568(21)00172-0

2. Tatangelo G, Watts J, Lim K, et al. The Cost of Osteoporosis, Osteopenia, and Associated Fractures in Australia in 2017. *J Bone Miner Res*. Apr 2019;34(4):616-625. doi:10.1002/jbmr.3640

3. Holgate J, Kirmani S, Anand B. Virtual fracture clinic delivers British Orthopaedic Association compliance. *Ann R Coll Surg Engl*. Jan 2017;99(1):51-54. doi:10.1308/rcsann.2016.0255

4. Waters S, Edmondston SJ, Yates PJ, Gucciardi DF. Identification of factors influencing patient satisfaction with orthopaedic outpatient clinic consultation: A qualitative study. *Man Ther*. Sep 2016;25:48-55. doi:10.1016/j.math.2016.05.334

5. Wraighte PJ, Scammell BE. Principles of fracture healing. *Surgery (Oxford)*. 2006;24(6):198-207. doi:10.1383/surg.2006.24.6.198

6. Ferguson KB, McGlynn J, Jenkins P, Madeley NJ, Kumar CS, Rymaszewski L. Fifth metatarsal fractures - Is routine follow-up necessary? *Injury*. Aug 2015;46(8):1664-8. doi:10.1016/j.injury.2015.05.041

7. Jenkins PJ, Gilmour A, Murray O, et al. The Glasgow Fracture Pathway: a virtual clinic. *BJJ News*. 2014;(2)(March):22-24.

For peer review only - http://bmjopen.bmj.com/site/about/guidelines.xhtml

8. Khan SA, Asokan A, Handford C, Logan P, Moores T. How useful are virtual fracture clinics?: a systematic review. *Bone Jt Open*. Nov 1 2020;1(11):683-690. doi:10.1302/2633-1462.111.Bjo-2020-0107.R1

9. Chan A-W, Tetzlaff JM, Altman DG, et al. SPIRIT 2013 Statement: Defining Standard Protocol Items for Clinical Trials. *Annals of Internal Medicine*. 2013/02/05 2013;158(3):200-207. doi:10.7326/0003-4819-158-3-201302050-00583

10. Shaw M, Anderson T, Sinclair T, et al. rpavirtual: Key lessons in healthcare organisational resilience in the time of COVID-19. *Int J Health Plann Manage*. May 2022;37(3):1229-1237. doi:10.1002/hpm.3430

11. Harris PA, Taylor R, Minor BL, et al. The REDCap consortium: Building an international community of software platform partners. *Journal of Biomedical Informatics*. 2019/07/01/ 2019;95:103208. doi:https://doi.org/10.1016/j.jbi.2019.103208

12. Harris PA, Taylor R, Thielke R, Payne J, Gonzalez N, Conde JG. Research electronic data capture (REDCap)—A metadata-driven methodology and workflow process for providing translational research informatics support. *Journal of Biomedical Informatics*. 2009/04/01/ 2009;42(2):377-381. doi:https://doi.org/10.1016/j.jbi.2008.08.010

13. Novak CB, Williams MM, Conaty K. Evaluation of the Patient-Specific Functional Scale in hand fractures and dislocations. *Hand (New York, NY)*. 2015;10(1):85-87. doi:10.1007/s11552-014-9658-2

14. Mannberg Bäckman S, Stråt S, Ahlström S, Brodin N. Validity and sensitivity to change of the Patient Specific Functional Scale used during rehabilitation following proximal humeral fracture. *Disabil Rehabil*. 2016;38(5):487-92. doi:10.3109/09638288.2015.1044623

15. Stratford P, Gill C, Westaway M, Binkley J. Assessing Disability and Change on Individual Patients: A Report of a Patient Specific Measure. *Physiotherapy Canada*. 1995/10/01 1995;47(4):258-263. doi:10.3138/ptc.47.4.258

16. Norman R, Mulhern B, Lancsar E, et al. The Use of a Discrete Choice Experiment Including Both Duration and Dead for the Development of an EQ-5D-5L Value Set for Australia. *Pharmacoeconomics*. Apr 2023;41(4):427-438. doi:10.1007/s40273-023-01243-0

17. Sjetne IS, Bjertnaes OA, Olsen RV, Iversen HH, Bukholm G. The Generic Short Patient Experiences Questionnaire (GS-PEQ): identification of core items from a survey in Norway. *BMC Health Serv Res*. Apr 21 2011;11:88. doi:10.1186/1472-6963-11-88

For peer review only - http://bmjopen.bmj.com/site/about/guidelines.xhtml

18. Bijur PE, Silver W, Gallagher EJ. Reliability of the visual analog scale for measurement of acute pain. *Acad Emerg Med*. Dec 2001;8(12):1153-7. doi:10.1111/j.1553-2712.2001.tb01132.x

19. Cleveland Clinic. Sample Size Estimation in Clinical Research. Department of Quantitative Health Services. Accessed 02 September, 2022.

20. Navarro-Pujalte E, Gacto-Sanchez M, Montilla-Herrador J, Escolar-Reina P, Angeles Franco-Sierra M, Medina-Mirapeix F. Sensitivity to change of mobility measures in musculoskeletal conditions on lower extremities in outpatient rehabilitation settings. *Disabil Rehabil*. Jun 2019;41(11):1268-1274. doi:10.1080/09638288.2018.1424948

21. Abbott JH, Schmitt J. Minimum important differences for the patient-specific functional scale, 4 region-specific outcome measures, and the numeric pain rating scale. *J Orthop Sports Phys Ther*. Aug 2014;44(8):560-4. doi:10.2519/jospt.2014.5248

22. Pathak A, Wilson R, Sharma S, et al. Measurement Properties of the Patient-Specific Functional Scale and Its Current Uses: An Updated Systematic Review of 57 Studies Using COSMIN Guidelines. *J Orthop Sports Phys Ther*. May 2022;52(5):262-275. doi:10.2519/jospt.2022.10727

23. Committee for Medicinal Products for Human Use (CHMP). Guideline on the Choice of the Non-inferiority Margin (EMA) (2005).

24. Husereau D, Drummond M, Augustovski F, et al. Consolidated Health Economic Evaluation Reporting Standards 2022 (CHEERS 2022) Statement: Updated Reporting Guidance for Health Economic Evaluations. *Value in Health*. 2022/01/01/ 2022;25(1):3-9. doi:https://doi.org/10.1016/j.jval.2021.11.1351

25. Petrou S, Gray A. Economic evaluation alongside randomised controlled trials: design, conduct, analysis, and reporting. *BMJ*. 2011;342:d1548. doi:10.1136/bmj.d1548

26. Braun V, Clarke V. Using thematic analysis in psychology. *Qualitative Research in Psychology*. 2006/01/01 2006;3(2):77-101. doi:10.1191/1478088706qp063oa

27. Safety monitoring and reporting in clinical trials involving therapeutic goods (Commonwealth of Australia, Canberra) (2016).

28. Management of Data and Information in Research: A guide supporting the Australian Code for the Responsible Conduct of Research (Commonwealth of Australia, Canberra) (2018).

For peer review only - http://bmjopen.bmj.com/site/about/guidelines.xhtml

For peer review only

For peer review only - http://bmjopen.bmj.com/site/about/guidelines.xhtml

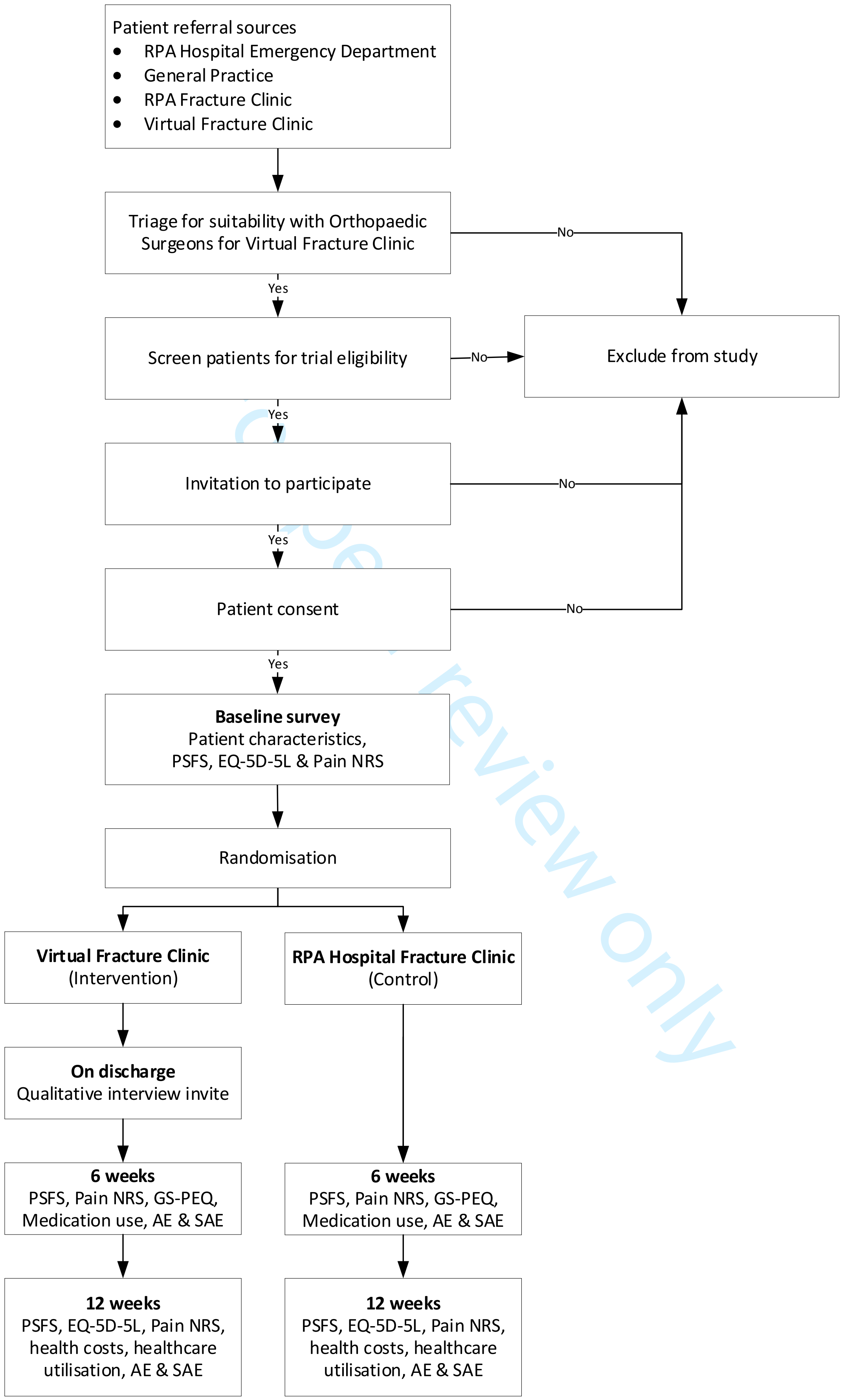

Figure 1: Trial design

For peer review only - http://bmjopen.bmj.com/site/about/guidelines.xhtml

Milestones	Enrolment	Baseline	6-weeks	12-weeks
Participant consent	✓			
Inclusion / Exclusion criteria	✓			
**Outcomes measures**				
Baseline patient characteristics		✓		
Patient-Specific Functional Scale		✓	✓	✓
EuroQol 5D 5L		✓		✓
Pain Numerical Rating Scale		✓	✓	✓
Generic Short Patient Experiences Questionnaire			✓	
Health costs				✓
Healthcare utilisation				✓
Medication use			✓	
Adverse events & serious adverse events			✓	✓

Table 1: Outcome measures

For peer review only

For peer review only - http://bmjopen.bmj.com/site/about/guidelines.xhtml

STANDARD PROTOCOL ITEMS: RECOMMENDATIONS FOR INTERVENTIONAL TRIALS

SPIRIT 2013 Checklist: Recommended items to address in a clinical trial protocol and related documents*

Section/item	ItemNo	Description	Page & section of item in protocol
**Administrative information**			
Title	1	Descriptive title identifying the study design, population, interventions, and, if applicable, trial acronym	1 – Front Page
Trial registration	2a	Trial identifier and registry name. If not yet registered, name of intended registry	3 – Trial registration number<br>6 – Design
	2b	All items from the World Health Organization Trial Registration Data Set	Added as appendix
Protocol version	3	Date and version identifier	16 – Ethics and dissemination
Funding	4	Sources and types of financial, material, and other support	16 – Funding statement
Roles and responsibilities	5a	Names, affiliations, and roles of protocol contributors	1 – Front Page<br>16 – Author's contributions
	5b	Name and contact information for the trial sponsor	N/A
	5c	Role of study sponsor and funders, if any, in study design; collection, management, analysis, and interpretation of data; writing of the report; and the decision to submit the report for publication, including whether they will have ultimate authority over any of these activities	16 – Funding statement

For peer review only - http://bmjopen.bmj.com/site/about/guidelines.xhtml

	5d	Composition, roles, and responsibilities of the coordinating centre, steering committee, endpoint adjudication committee, data management team, and other individuals or groups overseeing the trial, if applicable (see Item 21a for data monitoring committee)	14 – Data monitoring<br>16 – Ethics and dissemination
**Introduction**			
Background and rationale	6a	Description of research question and justification for undertaking the trial, including summary of relevant studies (published and unpublished) examining benefits and harms for each intervention	3 – Introduction
	6b	Explanation for choice of comparators	3 – Introduction
Objectives	7	Specific objectives or hypotheses	3 – Introduction
Trial design	8	Description of trial design including type of trial (eg, parallel group, crossover, factorial, single group), allocation ratio, and framework (eg, superiority, equivalence, noninferiority, exploratory)	6 – Design
**Methods: Participants, interventions, and outcomes**			
Study setting	9	Description of study settings (eg, community clinic, academic hospital) and list of countries where data will be collected. Reference to where list of study sites can be obtained	6 – Setting
Eligibility criteria	10	Inclusion and exclusion criteria for participants. If applicable, eligibility criteria for study centres and individuals who will perform the interventions (eg, surgeons, psychotherapists)	6 – Eligibility criteria
Interventions	11a	Interventions for each group with sufficient detail to allow replication, including how and when they will be administered	8 – Interventions

For peer review only - http://bmjopen.bmj.com/site/about/guidelines.xhtml

	11b	Criteria for discontinuing or modifying allocated interventions for a given trial participant (eg, drug dose change in response to harms, participant request, or improving/worsening disease)	8 – Interventions
	11c	Strategies to improve adherence to intervention protocols, and any procedures for monitoring adherence (eg, drug tablet return, laboratory tests)	8 – Interventions
	11d	Relevant concomitant care and interventions that are permitted or prohibited during the trial	15 – Ancillary and post-trial care
Outcomes	12	Primary, secondary, and other outcomes, including the specific measurement variable (eg, systolic blood pressure), analysis metric (eg, change from baseline, final value, time to event), method of aggregation (eg, median, proportion), and time point for each outcome. Explanation of the clinical relevance of chosen efficacy and harm outcomes is strongly recommended	9 – Outcomes
Participant timeline	13	Time schedule of enrolment, interventions (including any run-ins and washouts), assessments, and visits for participants. A schematic diagram is highly recommended (see Figure)	Table 1 added as appendix
Sample size	14	Estimated number of participants needed to achieve study objectives and how it was determined, including clinical and statistical assumptions supporting any sample size calculations	10 – Sample size
Recruitment	15	Strategies for achieving adequate participant enrolment to reach target sample size	6 – Eligibility criteria

Methods: Assignment of interventions (for controlled trials)

Allocation:

For peer review only - http://bmjopen.bmj.com/site/about/guidelines.xhtml

Sequence generation	16a	Method of generating the allocation sequence (eg, computer-generated random numbers), and list of any factors for stratification. To reduce predictability of a random sequence, details of any planned restriction (eg, blocking) should be provided in a separate document that is unavailable to those who enrol participants or assign interventions	6 – Eligibility criteria
Allocation concealment mechanism	16b	Mechanism of implementing the allocation sequence (eg, central telephone; sequentially numbered, opaque, sealed envelopes), describing any steps to conceal the sequence until interventions are assigned	6 – Eligibility criteria
Implementation	16c	Who will generate the allocation sequence, who will enrol participants, and who will assign participants to interventions	6 – Eligibility criteria
Blinding (masking)	17a	Who will be blinded after assignment to interventions (eg, trial participants, care providers, outcome assessors, data analysts), and how	11 – Blinding
	17b	If blinded, circumstances under which unblinding is permissible, and procedure for revealing a participant's allocated intervention during the trial	N/A
**Methods: Data collection, management, and analysis**			
Data collection methods	18a	Plans for assessment and collection of outcome, baseline, and other trial data, including any related processes to promote data quality (eg, duplicate measurements, training of assessors) and a description of study instruments (eg, questionnaires, laboratory tests) along with their reliability and validity, if known. Reference to where data collection forms can be found, if not in the protocol	9 – Outcomes<br>11 – Data collection methods

For peer review only - http://bmjopen.bmj.com/site/about/guidelines.xhtml

	18b	Plans to promote participant retention and complete follow-up, including list of any outcome data to be collected for participants who discontinue or deviate from intervention protocols	12 – Statistical methods<br>12 – Secondary analysis<br>13 – Cost-effectiveness analysis
Data management	19	Plans for data entry, coding, security, and storage, including any related processes to promote data quality (eg, double data entry; range checks for data values). Reference to where details of data management procedures can be found, if not in the protocol	11 – Data management
Statistical methods	20a	Statistical methods for analysing primary and secondary outcomes. Reference to where other details of the statistical analysis plan can be found, if not in the protocol	12 – Statistical methods
	20b	Methods for any additional analyses (eg, subgroup and adjusted analyses)	13 – Cost-effectiveness analysis<br>13 – Qualitative interview analysis
	20c	Definition of analysis population relating to protocol non-adherence (eg, as randomised analysis), and any statistical methods to handle missing data (eg, multiple imputation)	12 – Statistical methods<br>12 – Secondary analysis<br>13 – Cost-effectiveness analysis
**Methods: Monitoring**			
Data monitoring	21a	Composition of data monitoring committee (DMC); summary of its role and reporting structure; statement of whether it is independent from the sponsor and competing interests; and reference to where further details about its charter can be found, if not in the protocol. Alternatively, an explanation of why a DMC is not needed	14 – Data monitoring
	21b	Description of any interim analyses and stopping guidelines, including who will have access to these interim results and make the final decision to terminate the trial	N/A

For peer review only - http://bmjopen.bmj.com/site/about/guidelines.xhtml

Harms	22	Plans for collecting, assessing, reporting, and managing solicited and spontaneously reported adverse events and other unintended effects of trial interventions or trial conduct	9 – Outcomes<br>14 – Harms
Auditing	23	Frequency and procedures for auditing trial conduct, if any, and whether the process will be independent from investigators and the sponsor	14 – Auditing
**Ethics and dissemination**			
Research ethics approval	24	Plans for seeking research ethics committee/institutional review board (REC/IRB) approval	16 – Ethics and dissemination
Protocol amendments	25	Plans for communicating important protocol modifications (eg, changes to eligibility criteria, outcomes, analyses) to relevant parties (eg, investigators, REC/IRBs, trial participants, trial registries, journals, regulators)	16 – Ethics and dissemination
Consent or assent	26a	Who will obtain informed consent or assent from potential trial participants or authorised surrogates, and how (see Item 32)	14 – Consent or assent
	26b	Additional consent provisions for collection and use of participant data and biological specimens in ancillary studies, if applicable	N/A
Confidentiality	27	How personal information about potential and enrolled participants will be collected, shared, and maintained in order to protect confidentiality before, during, and after the trial	15 – Access to data
Declaration of interests	28	Financial and other competing interests for principal investigators for the overall trial and each study site	17 – Competing interests statement

For peer review only - http://bmjopen.bmj.com/site/about/guidelines.xhtml

Access to data	29	Statement of who will have access to the final trial dataset, and disclosure of contractual agreements that limit such access for investigators	15 – Access to data
Ancillary and post-trial care	30	Provisions, if any, for ancillary and post-trial care, and for compensation to those who suffer harm from trial participation	15 – Ancillary and posts-trial care
Dissemination policy	31a	Plans for investigators and sponsor to communicate trial results to participants, healthcare professionals, the public, and other relevant groups (eg, via publication, reporting in results databases, or other data sharing arrangements), including any publication restrictions	15 – Dissemination policy
	31b	Authorship eligibility guidelines and any intended use of professional writers	15 – Dissemination policy
	31c	Plans, if any, for granting public access to the full protocol, participant-level dataset, and statistical code	15 – Access to data
**Appendices**			
Informed consent materials	32	Model consent form and other related documentation given to participants and authorised surrogates	N/A
Biological specimens	33	Plans for collection, laboratory evaluation, and storage of biological specimens for genetic or molecular analysis in the current trial and for future use in ancillary studies, if applicable	N/A

*It is strongly recommended that this checklist be read in conjunction with the SPIRIT 2013 Explanation & Elaboration for important clarification on the items. Amendments to the protocol should be tracked and dated. The SPIRIT checklist is copyrighted by the SPIRIT Group under the Creative Commons "Attribution-NonCommercial-NoDerivs 3.0 Unported" license.

For peer review only - http://bmjopen.bmj.com/site/about/guidelines.xhtml

World Health Organization

International Clinical Trials Registry Platform
Search Portal

Home | Advanced Search | List By | Search Tips | UTN | ICTRP website | REGTRAC | Contact us

Main

Note: This record shows only 22 elements of the WHO Trial Registration Data Set. To view changes that have been made to the source record, or for additional information about this trial, click on the URL below to go to the source record in the primary register.

**Register:**	ANZCTR
**Last refreshed on:**	12 September 2023
**Main ID:**	ACTRN12623000934640
**Date of registration:**	29/08/2023
**Prospective Registration:**	Yes
**Primary sponsor:**	RPA Virtual Hospital
**Public title:**	RECITAL: Evaluating a virtual clinic for people with simple fractures
**Scientific title:**	Effects of virtual fractuRE Clinic care compared with In-person fracture clinic care on physical function in people with simple fractures: a non-inferiority randomised TriAL (RECITAL)
**Date of first enrolment:**	04/09/2023
**Target sample size:**	312
**Recruitment status:**	Not yet recruiting
**URL:**	https://anzctr.org.au/ACTRN12623000934640.aspx
**Study type:**	Interventional
**Study design:**	Purpose: Treatment; Allocation: Randomised controlled trial; Masking: Blinded (masking used);Assignment: Parallel;Type of endpoint: Safety/efficacy;
**Phase:**	Not Applicable

Countries of recruitment

Australia

Contacts

**Name:**	Mr Min Jiat Teng	**Name:**	Mr Min Jiat Teng
**Address:**	RPA Virtual Hospital Level 9, King George V Building Missenden Road Camperdown NSW 2050 Australia	**Address:**	RPA Virtual Hospital Level 9, King George V Building Missenden Road Camperdown NSW 2050 Australia
**Telephone:**	+61 421 398 669	**Telephone:**	+61 421 398 669
**Email:**	min.teng@health.nsw.gov.au	**Email:**	min.teng@health.nsw.gov.au
**Affiliation:**		**Affiliation:**	

Key inclusion & exclusion criteria

Inclusion criteria: a. Acute (<6 weeks) simple fractures such as base of fifth metatarsal (foot), Weber A (ankle), Mason I radial head (elbow) or clavicle (collar bone).
b. Aged 18 years or older,
c. The condition can be managed using removable orthoses including tapes and bandages,
d. Conditions in point (a) that are deemed appropriate for virtual management by the orthopaedic doctor,
e. Patient has access to a phone and an active telephone number,
f. Patient is within New South Wales at the time of consult,
g. Patient is willing to participate and comply with the study requirements,
h. A radiology scan showing or reporting the injury mentioned in point (a).
Exclusion criteria: a. Patients with complex or significantly displaced fracture, including pathological, open, unstable or spinal fractures,
b. Patients requiring a cast or surgical management,
c. Neurovascular concerns,
d. Conditions not managed by RPA Hospital Orthopaedics Department,
e. Patients who are unable to attend the in-person fracture clinic within the recommended follow-up time,
f. Patients who opt-out of this study.

Age minimum: 18 Years
Age maximum: No limit
Gender: Both males and females

Health Condition(s) or Problem(s) studied

Acute (<6weeks) simple fractures that can be managed conservatively, including non-displaced/minimally displaced limb fractures or injuries.;
Acute (<6weeks) simple fractures that can be managed conservatively, including non-displaced/minimally displaced limb fractures or injuries.

Musculoskeletal - Other muscular and skeletal disorders

Injuries and Accidents - Fractures

Intervention(s)

Participants in the intervention group will receive care virtually.

Patients will be provided a follow-up appointment at the virtual fracture clinic within 5 days after their referral is received. Patients will be sent an email with the details of their appointment date/time, a Zoom link for the virtual consult, contact details of the clinic, and the respective fracture management fact sheet. The RPA Virtual Hospital's fact sheets explain the clinical condition, expectation of the recovery, early rehabilitation exercises, activity limitations, and clear information on care escalation. These fact sheets were adapted with permission from the Royal Melbourne Hospital, and were approved by the RPA Virtual Hospital's Consumer Group.

All patients will be offered a video-consult with a physiotherapist unless they choose to have their review via phone. During the virtual consult, the physiotherapist will conduct a subjective and objective assessment, discuss the x-ray findings and provide a management plan. Patients will be offered the opportunity to ask questions prior to ending the consultation. The anticipated duration of the consult sessions will be approximately 30 minutes. An email summary of the consultation, along with the follow-up appointment details will be sent to the patient. A Physitrack link for tailored exercise videos and instructions may also be included in this email. Patients will be offered a follow-up virtual appointment at 2- and 6-weeks post-fracture, or based on clinical need. Patients can contact the physiotherapist via phone or email during business hours if they have any concerns during their care period. Most patients will be discharged at 6-weeks post-fracture if they have no other concerns. We will monitor patient adherence by the

Primary Outcome(s)

Physical function outcomes will be assessed using the Patient-Specific Functional Scale (PSFS)[Baseline, 6 and 12 weeks (primary timepoint) post-randomisation]

Secondary Outcome(s)

Number of patients requiring surgery will be measured through the electronic medical records.[12 weeks post-randomisation]

Adverse Events will be measured using a patient survey designed specifically for this study, safety reports from the electronic medical records (eMR), NSW Health Incident Management System (ims+).[6 and 12 weeks post-randomisation]

Pain will be measured using the 0-10 Numerical Rating Scale (NRS)[Baseline, 6 and 12 weeks post-randomisation]

For peer review only - http://bmjopen.bmj.com/site/about/guidelines.xhtml

Cost borne by the healthcare provider will be measured through the electronic medical records, Sydney Local Health District Targeted Activity and Reporting System (STARS App) Dashboard, and the hospital's performance, data and finance departments to obtain the healthcare appointment duration, healthcare provider's hourly rate, any health services utilisation and corresponding cost (including but not limited to outpatient, inpatient, emergency department, pharmacy, radiology, pathology and primary care), any infrastructure setup and maintenance cost, managerial and administrative overhead. [12 weeks post-randomisation]

Health-related quality of life will be assessed using the EuroQol 5D 5L (EQ-5D-5L)[Baseline and 12 weeks post-randomisation]

Medication use will be measured using a patient survey designed specifically for this study to assess the name and dose of prescription or over the counter medication for their injury. [6 weeks post-randomisation]

Cost borne by patients will be measured through the patient survey designed specifically for this study.[12 weeks post-randomisation]

Patient-reported experience will be measured using the Generic Short Patient Experiences Questionnaire (GS-PEQ)[6 weeks post-randomisation]

Healthcare utilisation will be measured using a patient survey designed specifically for this study to assess the number of other healthcare appointments for management of their injury. The research team will also ascertain if the patients utilised any other healthcare services through the patient's electronic medical records.[12 weeks post-randomisation]

Emergency Department re-presentations will be measured through the electronic medical records.[12 weeks post-randomisation]

Serious Adverse Events will be measured using a patient survey designed specifically for this study, safety reports from the electronic medical records (eMR), NSW Health Incident Management System (ims+).[6 and 12 weeks post-randomisation]

Secondary ID(s)

None

Source(s) of Monetary Support

Sydney Research – Clinician Researcher Scholarship

NHMRC 2022 MRFF Clinician Researchers - Nurses Midwives and Allied Health

Secondary Sponsor(s)

Ethics review

Status: Approved
Approval date: 14/07/2023
Contact:
Sydney Local Health District Human Research Ethics Committee (HREC) - RPAH

Results

Results available:
Date Posted:
Date Completed:
URL:

Disclaimer: Trials posted on this search portal are not endorsed by WHO, but are provided as a service to our users. In no event shall the World Health Organization be liable for any damages arising from the use of the information linked to in this section. None of the information obtained through use of the search portal should in any way be used in clinical care without consulting a physician or licensed health professional. WHO is not responsible for the accuracy, completeness and/or use made of the content displayed for any trial record.

Copyright - World Health Organization - Version 3.6 - Version history

For peer review only

BMJ Open

RECITAL: A non-inferiority randomised control trial evaluating a virtual fracture clinic compared with in-person care for people with simple fractures

Journal:	*BMJ Open*
Manuscript ID	bmjopen-2023-080800.R1
Article Type:	Protocol
Date Submitted by the Author:	20-Dec-2023
Complete List of Authors:	Teng, Min Jiat; The University of Sydney Faculty of Medicine and Health, Institute for Musculoskeletal Health, Sydney School of Public Health; Sydney Local Health District, RPA Virtual Hospital<br>Zadro, Joshua; The University of Sydney Faculty of Medicine and Health, Institute for Musculoskeletal Health, Sydney School of Public Health<br>Pickles, Kristen; The University of Sydney Faculty of Medicine and Health, Sydney Health Literacy Lab, Sydney School of Public Health<br>Copp, Tessa; The University of Sydney Faculty of Medicine and Health, Sydney Health Literacy Lab, Sydney School of Public Health<br>Shaw, Miranda; Sydney Local Health District, RPA Virtual Hospital<br>Khoudair, Isabella; Sydney Local Health District, RPA Virtual Hospital<br>Horsley, Mark; Sydney Local Health District, Department of Orthopaedic Surgery, Royal Prince Alfred Hospital<br>Warnock, Benjamin; Sydney Local Health District, RPA Virtual Hospital<br>Hutchings, Owen; Sydney Local Health District, RPA Virtual Hospital<br>Petchell, Jeffrey; Sydney Local Health District, Department of Orthopaedic Surgery, Royal Prince Alfred Hospital<br>Ackerman, Ilana; Monash University, School of Public Health and Preventive Medicine<br>Drayton, Alison; Consumer representative<br>Liu, Rong; Sydney Local Health District, RPA Virtual Hospital; The George Institute for Global Health, University of New South Wales<br>Maher, Christopher; The University of Sydney Faculty of Medicine and Health, Institute for Musculoskeletal Health, Sydney School of Public Health<br>Traeger, Adrian; The University of Sydney Faculty of Medicine and Health, Institute for Musculoskeletal Health, Sydney School of Public Health
**Primary Subject Heading**:	Health services research
Secondary Subject Heading:	Health economics, Qualitative research
Keywords:	Telemedicine < BIOTECHNOLOGY & BIOINFORMATICS, ORTHOPAEDIC & TRAUMA SURGERY, Randomized Controlled Trial, Protocols & guidelines < HEALTH SERVICES ADMINISTRATION & MANAGEMENT, Patient Reported Outcome Measures

SCHOLARONE™
Manuscripts

RECITAL: A non-inferiority randomised control trial evaluating a virtual fracture clinic compared with in-person care for people with simple fractures

Min Jiat Teng[1,3], Joshua R Zadro[1], Kristen Pickles[2], Tessa Copp[2], Miranda Shaw[3], Isabella Khoudair[3], Mark Horsley[4], Benjamin Warnock[3], Owen Hutchings[3], Jeffrey Petchell[4], Ilana Ackerman[5], Alison Drayton[6], Rong Liu[3,7], Chris G Maher[1], and Adrian C Traeger[1]

[1]The Institute for Musculoskeletal Health, The University of Sydney and Sydney Local Health District, Gadigal Country, Sydney, New South Wales, Australia

[2]University of Sydney, Sydney Health Literacy Lab, School of Public Health, Faculty of Medicine and Health, Sydney, New South Wales, Australia

[3]RPA Virtual Hospital, Sydney Local Health District, Sydney, New South Wales, Australia

[4]Department of Orthopaedic Surgery, Royal Prince Alfred Hospital, Sydney Local Health District, Sydney, New South Wales, Australia

[5]School of Public Health and Preventative Medicine, Monash University, Melbourne, Victoria, Australia

[6]Consumer representative

[7]The George Institute for Global Health, University of New South Wales, Sydney, New South Wales, Australia

CORRESPONDENCE

Min Jiat Teng
Min.Teng@health.nsw.gov.au
The Institute for Musculoskeletal Health
Level 10N, King George V Building, Royal Prince Alfred Hospital
PO Box M179, Missenden Road Camperdown, NSW, AUS 2050

For peer review only - http://bmjopen.bmj.com/site/about/guidelines.xhtml

KEYWORDS

Telemedicine, orthopaedic, randomised controlled trial, protocol, Patient-Specific Functional Scale

WORD COUNT: 3937 words

For peer review only

For peer review only - http://bmjopen.bmj.com/site/about/guidelines.xhtml

ABSTRACT

INTRODUCTION

Most simple undisplaced fractures can be managed without surgery by immobilising the limb with a splint, prescribing medication for pain, and providing advice and early rehabilitation. Recent systematic reviews based on retrospective observational studies have reported that virtual fracture clinics can deliver follow-up care that is safe and cost-effective. However, no randomised controlled trial has investigated if a virtual fracture clinic can provide non-inferior physical function outcomes compared to an in-person clinic for patients with simple fractures.

METHODS AND ANALYSIS

312 participants will be recruited from two metropolitan hospitals located in Sydney, Australia. Adult patients will be eligible if they have an acute simple fracture that can be managed with a removable splint, and is deemed appropriate for follow-up at either the virtual or in-person fracture clinic by an orthopaedic doctor. Patients will not be eligible if they have a complex fracture that requires a cast or surgery. Eligible participants will be randomised to receive their follow-up care either at the virtual or the in-person fracture clinic. Participants at the virtual fracture clinic will be reviewed within five days of receiving a referral through video calls with a physiotherapist. Participants at the in-person fracture clinic will be reviewed within seven to ten days of receiving a referral with an orthopaedic doctor. The primary outcome will be the patient's function measured using the Patient-Specific Functional Scale at 12 weeks. Secondary outcomes will include health-related quality of life, patient-reported experiences, pain, health cost, healthcare utilisation, medication use, adverse events, emergency department re-presentations and surgery.

ETHICS AND DISSEMINATION

The study has been approved by the Sydney Local Health District Ethics Review Committee (RPAH Zone) (X23-0200 & 2023/ETH01038). The trial results will be submitted for publication in a reputable international journal and will be presented at professional conferences.

TRIAL REGISTRATION NUMBER

For peer review only - http://bmjopen.bmj.com/site/about/guidelines.xhtml

https://www.anzctr.org.au/Trial/Registration/TrialReview.aspx?ACTRN=12623000934640; ACTRN12623000934640

STRENGTH AND LIMITATIONS OF THIS STUDY

- Pragmatic clinical trial embedded within two existing fracture clinics at two urban hospitals
- Measures hospital-level outcomes as well as patient outcomes and experiences
- Blinding of therapist or participants is not possible, although participants are blinded to the study hypothesis
- Methods and results from this trial may inform the evaluation of other virtual musculoskeletal services
- Study may not be sufficiently powered to determine subgroup effects e.g. based on specific fracture diagnosis

For peer review only

For peer review only - http://bmjopen.bmj.com/site/about/guidelines.xhtml

INTRODUCTION

In 2019, there were 178 million new fractures reported globally, an increase of 33.4% since 1990.[1] In Australia, the treatment costs of osteoporosis-related fractures were estimated to be A$2.34 billion in 2017.[2] With increasing numbers of people requiring care for their fractures, the burden on outpatient fracture clinics has also increased, causing long clinic wait times, and productivity losses.[3, 4] The recent pandemic further strengthens the requirement for health system efficiency.

Most simple fractures, including minimally displaced fractures, can be managed conservatively without surgery. These stable fractures are managed with short-term immobilisation, advice, pain relieving medication, and early rehabilitation.[5] Traditional physical assessments at an outpatient clinic may not be required for conditions that have a clear prognosis and have been shown to recover well with conservative management.[6]

Published studies have shown that virtual fracture clinics can manage patients with simple fractures.[7] Patients receive advice and management through phone calls and written handouts, rather than attending the outpatient clinic in-person. Retrospective observational studies have reported that virtual fracture clinics are associated with good patient satisfaction, increased cost-efficiency for the hospital system, fewer adverse events and reduced presentations to in-person clinics.[8]

Despite a rise in virtual fracture clinics since the recent pandemic, robust evaluations of their safety, effectiveness and cost-effectiveness are lacking. A recent systematic review of 21 publications suggested that virtual fracture clinics could provide safe and cost-effective care to patients with acute fractures, though none of the included studies were randomised controlled trials.[8] It is currently unknown whether virtual fracture clinics produce non-inferior outcomes compared with in-person care for patients with simple fractures.

We have designed a clinical trial to evaluate the effectiveness of a virtual fracture clinic for patients with simple fractures. The primary outcome of this trial is physical function at 12-weeks follow-up, measured using the Patient-Specific Functional Scale (PSFS). Secondary outcomes include pain, quality of life, patient-reported experience measures, cost-effectiveness,

For peer review only - http://bmjopen.bmj.com/site/about/guidelines.xhtml

healthcare utilisation, medication use and safety. A qualitative sub-study will be conducted to explore the experiences, feelings and expectations of patients who use the virtual fracture clinic.

METHODS AND ANALYSIS

Design

The Fracture Clinic Trial (RECITAL) is a prospective two-arm, parallel group randomised controlled trial, using a non-inferiority design with nested economic and process evaluations. We chose a non-inferiority randomised controlled trial design as both study groups are existing hospital services, and the virtual fracture clinic is expected to have outcomes that are at least no worse than the in-person fracture clinic. This trial has been prospectively registered with the Australian New Zealand Clinical Trials Registry (ACTRN12623000934640). This document describes the trial protocol according the Standard Protocol Items: Recommendations for Interventional Trials (SPIRIT) 2013 Statement.[9] Recruitment began in September 2023, with the final data collection expected to occur in November 2025.

Setting

RECITAL will compare two existing models of care provided at two metropolitan public hospitals within Sydney Local Health District (SLHD) in New South Wales, Australia. The virtual fracture clinic (intervention group) is located at RPA Virtual Hospital (**rpa**virtual), while the in-person fracture clinic (control group) is situated at the Royal Prince Alfred (RPA) Hospital. **rpa**virtual is Australia's first virtual hospital established in February 2020 to enable patients to receive hospital-level care at home through virtual means (e.g. video calls or remote monitoring), rather than visiting the traditional hospital for their healthcare needs.[10]

Eligibility criteria

Patients referred to the virtual fracture clinic (e.g. from local Emergency Departments, General Practices, or the in-person fracture clinic) will be identified and screened by a virtual fracture clinic physiotherapist and an orthopaedic doctor to determine if the patient is suitable for either

For peer review only - http://bmjopen.bmj.com/site/about/guidelines.xhtml

model of care (virtual or in-person). The RECITAL study staff will contact the eligible patients to invite them into this study. Figure 1 illustrates the trial design.

Patients will be invited to participate if they meet the following criteria:

- Have an acute (<6 weeks old) simple fracture that can be managed using a removable orthoses (e.g. shoulder immobiliser, CAMboot or wrist splint)
- Aged ≥18 years
- Have a condition that is deemed appropriate for virtual management by an orthopaedic doctor
- Has access to a phone and will be within New South Wales at the time of consult
- Is willing to participate and comply with the study requirements
- Have a radiology scan or report to confirm the nature of the injury

Patients will be excluded if they have:

- Complex or significantly displaced fracture, including pathological, open, unstable or spinal fractures requiring a cast or surgical management
- Neurovascular concerns
- A condition not managed by RPA Hospital Orthopaedics Department
- Reported being unable to attend the in-person fracture clinic within the recommended follow-up time
- Opted out

People with any type of simple fracture that is deemed appropriate for virtual care will be eligible for the trial, to reflect usual practice. The most common types of fracture are expected to be base of fifth metatarsal, ankle Weber A, and Mason I radial head. Uncommon types of simple fracture could include greater tuberosity or clavicle. Patients who consent to participate and complete their baseline measures will be enrolled in this study. Informed consent (supplementary file) and study data will be collected and managed using the Research Electronic Data Capture (REDCap) tool hosted at SLHD.[11, 12] The randomisation schedule will be computer-generated using REDCap's randomisation model, and will be stratified in random blocks of 4, 6, 8 and 10 to ensure equal numbers in both groups and concealed allocation. A biostatistician not involved in this study will set up the allocation schedule and

For peer review only - http://bmjopen.bmj.com/site/about/guidelines.xhtml

upload it into REDCap. Only the biostatistician will be aware of the allocation to ensure concealment. The study coordinator will randomise the patients to the study groups. Participants randomised to the virtual fracture clinic who agree to participate in the qualitative sub-study will be purposively selected for an interview according to their age, employment status, tertiary education level, type of injury and discharge status. Selected participants will be contacted once they are discharged from the clinic to ensure their complete experience with the virtual fracture clinic is captured.

Interventions

Both study groups reflect current processes within existing clinics at the participating hospitals.

Virtual Fracture Clinic (VFC) (Intervention Group)

Patients randomised to the VFC (intervention) group will be contacted via phone and email to organise an initial follow-up with a physiotherapist; usually within 5 days after their referral is received. Patients are sent an email with their appointment details and a fracture management fact sheet. The fact sheet explains their clinical condition, expected recovery, early rehabilitation exercises, activity limitations, and information on care escalation. These fact sheets were adapted with permission from Royal Melbourne Hospital's Virtual Fracture Clinic.

All patients are offered a video-consultation with a physiotherapist unless they choose to have their review via phone. During the virtual consults, the physiotherapist conducts an assessment, discusses the x-ray findings, and provides a management plan. The virtual consult sessions are usually approximately 30 minutes. An email summary of the consultation, and follow-up appointment details are sent to the patient after the consultation. A Physitrack link may also be included in this email. Physitrack or PhysiApp is an internet-based program that allows patients to view videos of their prescribed exercises. Patients are usually offered a follow-up virtual appointment at 2- and 6-weeks post-fracture, or based on clinical need. Patients can contact the physiotherapist out of session if they have any concerns during their care period. Most patients are discharged from the VFC at 6-weeks post-fracture if there are no concerns. Patients will be supported with an interpreter, Aboriginal Cultural Support Team or with loaned

For peer review only - http://bmjopen.bmj.com/site/about/guidelines.xhtml

devices and data as required. For example, although many older patients (aged 60+) currently use the virtual service, a Digital Patient Navigator can assist patients and provide a smart phone with data so they attend their virtual clinic appointments. We will monitor patient adherence by the number of consults attended; and the number of ad hoc patient contacts via phone or email with the clinic.

In-person Fracture Clinic (Control Group)

Patients randomised to the in-person fracture clinic will be contacted via phone or email to provide a follow-up appointment. Appointments usually occur 7 to 10 days after the referral is received, based on the availability of the on-call orthopaedic doctor. Clinical management and subsequent follow-ups of the control group will be determined by the orthopaedic doctors at the in-person fracture clinic. Clinical management can include a physical assessment by a doctor, radiology scan, advice and exercises. A physiotherapist may be involved in the patient's care. Patients in the control group may receive written instructions about their recovery and exercises as per current processes. The in-person consult sessions are usually approximately 20 minutes. Current practice suggests that patients may attend the in-person fracture clinic once or twice within 6-weeks post-fracture. We will monitor patient adherence by the number of consults attended.

Staff at both study groups will be trained on the trial protocol and be regularly supported by study investigators to ensure adherence to the study.

Outcomes

The primary outcome for this study will be the participant's physical function assessed using the PSFS at 12-weeks. This self-reported tool has shown to be sensitive to change in patients with musculoskeletal problems, including simple fractures.[13-15] Participants list up to five functional tasks at baseline, and score their level of ability – 0 (unable to perform activity) to 10 (able to perform activity at the same level as before the injury). Scores for each activity will be summed and calculated as an average of the total possible score for the participant (determined by the number of identified activities). We will compare the PSFS average scores between

For peer review only - http://bmjopen.bmj.com/site/about/guidelines.xhtml

groups at 12-weeks as our primary measure. Table 1 summarises the outcome measures for this study.

Milestones	Enrolment	Baseline	6-weeks	12-weeks
Participant consent	✓			
Inclusion / Exclusion criteria	✓			
Outcomes measures				
Baseline patient characteristics		✓		
Patient-Specific Functional Scale		✓	✓	✓
EuroQol 5D 5L		✓		✓
Pain Numerical Rating Scale		✓	✓	✓
Generic Short Patient Experiences Questionnaire			✓	
Health costs				✓
Healthcare utilisation				✓
Medication use			✓	
Adverse events & serious adverse events			✓	✓
Emergency Department re-presentations				✓
Patients requiring surgery				✓

Table 1: Outcome measures

Secondary measures for this study will include:

- Health-related quality of life measure assessed using the EuroQol 5D 5L (EQ-5D-5L) at baseline and 12-weeks[16]

For peer review only - http://bmjopen.bmj.com/site/about/guidelines.xhtml

- Patient-reported experience measure assessed using the Generic Short Patient Experiences Questionnaire (GS-PEQ) at 6-weeks[17]
- Pain assessed using the 0-10 Numerical Rating Scale (NRS) at baseline, 6- and 12-weeks[18]
- Cost borne by the healthcare service, measured at 12-weeks. We will collect data from the electronic medical records (eMR), Sydney Local Health District Targeted Activity and Reporting System (STARS App) Dashboard, and the hospital's performance, data and finance departments to obtain the healthcare appointment duration, healthcare provider's hourly rate, any health services utilisation and corresponding cost (including but not limited to outpatient, inpatient, emergency department, pharmacy, radiology, pathology and primary care), any infrastructure setup and maintenance cost, managerial and administrative overhead
- Cost borne by patients, measured through the patient survey designed specifically for this study at 12-weeks
- Healthcare utilisation assessed using a survey designed specifically for this study at 12-weeks. The survey will collect the number of other healthcare appointments for management of their injury. We will also ascertain if the patients utilised any other healthcare services through the patient's eMR
- Medication use assessed using a survey designed specifically for this study to assess the name and dose of prescription or over the counter medication for their injury at 6-weeks
- Adverse Events and Serious Adverse Events assessed using a survey designed specifically for this study at 6- and 12-weeks. We will also collect data from safety reports within the eMR, and the NSW Health Incident Management System (IMS+)
- Emergency Department re-presentations measured by reviewing the electronic medical records at 12-weeks. This information may also be reported in the healthcare utilisation survey and the adverse/serious adverse event survey

For peer review only - http://bmjopen.bmj.com/site/about/guidelines.xhtml

- Number of patients requiring surgery measured by reviewing the electronic medical records at 12-weeks. This information may also be reported in the healthcare utilisation survey and the adverse/serious adverse event survey

Sample size

A total sample of 312 participants will provide 90% power to detect a non-inferiority margin of 0.7 points on the 11-point PSFS with a 10% loss to follow-up, a standard deviation of 2.0, α of 5%, and a correlation score between baseline and final scores of 0.5 at 12-weeks.[19] A negative between-group difference of ≤0.7 points will indicate that the virtual fracture clinic is non-inferior to the in-person fracture clinic.

We chose a standard deviation of 2.0 as this is between the mean value of the standard deviation for the PSFS at follow-up in published studies that range from 1.7[20], 2.1[21] and 2.2[13]. The minimal important difference (MID) for the PSFS ranges from 1.3 (small change) to 2.7 (large change).[21, 22] Guidelines suggest using a non-inferiority margin of 50% (or less preferably) of the treatment effect of standard care vs placebo.[23] Thus, we chose a between-group non-inferiority margin of 0.7 (50% of the MID of 1.3).

Blinding

The participants, therapists and assessors will not be blinded. The surveys administered during this trial are self-assessments completed by patients directly in REDCap who will be blinded to the study hypothesis. If required, an independent blinded assessor may contact the patient to assist them with completing their surveys.

Data collection methods

Patients will receive a unique link via email or phone message to complete all their surveys directly in REDCap. Patients will receive an email or phone message 2 days prior to each milestone, reminding them to complete their respective surveys. Two reminders followed by a phone call will be provided to patients who do not complete their surveys by the respective

For peer review only - http://bmjopen.bmj.com/site/about/guidelines.xhtml

milestone. The treating clinicians may remind the participants to complete their surveys during their routine clinical reviews. The clinicians will not be able to complete, nor alter the results from the surveys. If requested, paper copies of the surveys may be sent to participants with their responses transcribed verbatim into REDCap by an independent blinded assessor not involved in this study.

Data management

All study data will be collected, logged and stored within SLHD's REDCap server. REDCap functions such as adding a field note (a brief descriptor of the question or answer), auto-calculations, and using data validation functions will be used to ensure data quality. The 'required' field is also used to ensure participants complete the mandatory questions prior to submitting the survey. The questionnaires will be tested by clinicians and patients prior to implementation. The research team will have access through a personal login and password.

Statistical methods

An intention-to-treat analysis will be implemented after the database is cleaned and locked. Separate analyses will be conducted on each outcome. Descriptive statistics will be used for patient demographics and clinical characteristics. Categorical variables will be described with frequencies (%), and continuous variables will be described with means and standard deviations. Data will be analysed using STATA version 14 statistical software (StataCorp, College Station, TX) or R version 4.2.1 (R Foundation for Statistical Computing, Vienna, Austria. https://www.R-project.org/).

Primary analysis

Non-inferiority trials assess whether an intervention outcome is not clinically worse than a control. The PSFS score at 12-weeks post-randomisation is the primary outcome in this study and we have prospectively defined a non-inferiority margin (ΔT) of -0.7 points, which is the maximum difference we are prepared to tolerate and still consider virtual care not to be clinically inferior to in-person care. The null hypothesis is therefore that a difference of greater than ΔT

For peer review only - http://bmjopen.bmj.com/site/about/guidelines.xhtml

exists in favour of in-person care (H0: $\Delta \leq -\Delta T$). This will be assessed by creating a 95% confidence interval, which should be entirely above the non-inferiority margin for the intervention to be declared non-inferior. The PSFS score will be compared between treatment groups as the dependent variable in a generalised linear regression model for the primary analysis adjusting for baseline PSFS variables. The treatment difference will be based on the estimate of adjusted means and 95% confidence intervals.

Secondary analysis

Secondary clinical outcomes will be analysed using logistic regression for binary outcomes and linear regression for continuous outcomes. Results from the analyses will be presented as point estimates with 95% confidence intervals. Baseline scores will be included in the model to increase statistical precision. If more than 5% of data are missing, then imputation techniques may be considered.

Cost-effectiveness analysis

The economic evaluation will estimate the difference in the cost of resource inputs used by participants in the two arms of the trial, allowing comparisons to be made between the two models of care.

We will conduct a cost-effectiveness analysis to estimate the incremental-effectiveness ratios (ICERs) defined as: [cost of the virtual care - cost of in-person practice]/[effectiveness of the virtual care - effectiveness of in-person practice]. The effectiveness outcomes include PSFS, ED visit, rehospitalisation, and quality-adjusted life-years (QALYs). Costs for resource inputs will largely be derived from available local and national sources and estimated in line with best practice. Primary research using established accounting methods may also be required to estimate unit costs. Costs will be standardised to current prices where possible. The EQ-5D-5L outcomes will be used to generate QALYs, and the responses will be compared to the national Australian value set for the EQ-5D-5L.[16] Multiple imputation methods will be used to impute missing data and avoid biases associated with complete case analysis.

To estimate the uncertainty of ICERs, bootstrapping will be used to resample corresponding costs and effectiveness that will be observed in RECITAL, and the distribution of ICERs

For peer review only - http://bmjopen.bmj.com/site/about/guidelines.xhtml

calculated from all resamples will be plotted on a cost-effectiveness plane. Subgroup analysis will be carried out to assess the equity impact of the interventions. One-way sensitivity analysis will be conducted around key cost variables. A cost-effectiveness acceptability curve (CEAC) will be plotted, which will provide information about the probability that an intervention is cost-effective, given the level of a decision maker's willingness to pay for each additional effectiveness outcome gain. The economic assessment method will adhere to the Consolidated Health Economic Evaluation Reporting Standards 2022 (CHEERS 2022).[24, 25]

Qualitative interview analysis

The thematic analysis will be based on Braun and Clarke's six-phase framework.[26] After the interview recordings are transcribed verbatim, the research team will independently annotate the transcripts to generate initial ideas and relevant phrases. A qualitative data analytic software (Nvivo) will be used to code and organise the data into themes. The topic guide may be modified between interviews to enable new emerging themes from the interviews to be explored more in depth with subsequent patients. The data will be reported according to the Consolidated Criteria for Reporting Qualitative Research (COREQ).

Data monitoring

Given the relatively low-risk nature of the intervention, a data safety and monitoring board will not be utilised in this study. The study coordinator will provide feedback (at least once per year) to the investigator team, which consists of orthopaedic doctors, senior researchers, hospital executives and a consumer.

Harms

Adverse and serious adverse events as defined by the National Health and Medical Research Council (NHMRC) will be monitored throughout this study.[27] Potential adverse events arising from this study include mis-diagnoses or missed diagnoses; emergency department re-presentations or surgical management of the fracture. All serious adverse events will be reported immediately to the investigator team and Human Research Ethics Committee.

For peer review only - http://bmjopen.bmj.com/site/about/guidelines.xhtml

Auditing

There are no planned audits for this study.

Consent or assent

The RECITAL study staff will contact all eligible patients to inform them about their follow-up options for their simple fractures using a standardised recruitment script. All patients who agree to have their follow-up care at one of the fracture clinics (virtual or in-person) will be invited to participate in this study. Study staff will send the patient a REDCap link via email or phone message for participants to view the study outline and requirements online, including the opportunity to download the Participant Information Sheet. If the patient agrees to participate, they will complete an e-Consent form within REDCap. Participants who choose not to participate in the RECITAL study will be able to choose their follow-up at the virtual or in-person fracture clinic. Only patients who have completed the e-Consent form will be enrolled into this study. Participants who complete all their surveys will be given A$50 to reimburse them for their time.

Access to data

Only clinicians providing care to the participants and the study coordinator will have access to the identifiable data. All other investigators of this study will have access to the de-identified data. As per NHMRC requirements, the research data from this study will be retained for 15 years from the end of the trial.[28] Study protocol will be made available upon reasonable request.

Ancillary and post-trial care

Study participants are free to engage with other treatment providers such as their general practitioner or outpatient physiotherapist during and after this study for the management of their injury. These costs will not be borne by the study. This study will capture these visits to other health care providers for the management of this injury through the healthcare utilisation survey.

For peer review only - http://bmjopen.bmj.com/site/about/guidelines.xhtml

Dissemination policy

The trial results will be submitted for publication in reputable international journals and will be presented at relevant professional conferences. The results will also be disseminated to the media. Authorship eligibility will align with the International Committee of Medical Journal Editors.

Patient and public involvement

AD is a co-investigator of this trial and has lived experiences at both fracture clinics investigated by this study. AD agreed that the research question was important, and has reviewed and provided feedback on all the study documents. Facts sheets used by the VFC have been approved by the **rpa**virtual Consumer Group. This study will investigate the experiences of participants through the Generic Short Patient Experiences Questionnaire and qualitative interviews. All participants can indicate on the consent form if they would like to receive the final study results.

CONCLUSION

This trial has been designed to be embedded in usual clinical practice to evaluate two existing models of care at two urban public hospitals. Results from this trial will inform patients, clinicians, hospitals, policy makers and health funders globally about the effectiveness of a virtual fracture clinic.

ETHICS AND DISSEMINATION

Study has been approved by the Sydney Local Health District Ethics Review Committee (RPAH Zone) (X23-0200 & 2023/ETH01038; 30 June 2023). Any amendments to the trial protocol will require approval from the trial's Steering Committee and the ethics committee prior to implementation. Recruitment will commence in October 2023 and is expected to complete by September 2026.

For peer review only - http://bmjopen.bmj.com/site/about/guidelines.xhtml

AUTHOR CONTRIBUTIONS

ACT, CGM and MJT conceived the idea for the trial. MJT drafted the manuscript and JRZ, CGM, ACT, IA, OH, MH, MJT, RL, KP, TC, IK, BW, MS, AD and JP contributed to the design of the study and critical review. All authors contributed to the design, intellectual content to the manuscript and approved the final version.

FUNDING STATEMENT

This work is supported by the National Health and Medical Research Council (NHMRC) 2022 Medical Research Future Fund (MRFF) Clinician Researchers – Nurses Midwives and Allied Health Grant Number 2022985 and Sydney Research 2022 Clinician Researcher Scholarship. The funding sources did not contribute to the trial design and will not have a role in the trial conduct, data analysis, interpretation, writing or reporting.

COMPETING INTERESTS STATEMENT

The following investigators (ACT, MS, OH, CGM, TC, KP, JZ, IA, RL, AD) have no conflict on interests to declare. The SLHD clinicians (MJT, IK, BW, MH, JP) may deliver care to participants in either study groups as part of their usual clinical role provided at the public hospitals. MJT will be conducting this study in partial fulfillment of the requirements of a Doctor of Philosophy (Medicine and Health) degree under the supervision AT, CGM, TC, KP and JZ.

REFERENCES

1. Wu A-M, Bisignano C, James SL, Abady GG, Abedi A, Abu-Gharbieh E, et al. Global, regional, and national burden of bone fractures in 204 countries and territories, 1990–2019: a systematic analysis from the Global Burden of Disease Study 2019. The Lancet Healthy Longevity. 2021;2(9):e580-e92.

For peer review only - http://bmjopen.bmj.com/site/about/guidelines.xhtml

2. Tatangelo G, Watts J, Lim K, Connaughton C, Abimanyi-Ochom J, Borgstrom F, et al. The Cost of Osteoporosis, Osteopenia, and Associated Fractures in Australia in 2017. J Bone Miner Res. 2019;34(4):616-25.

3. Holgate J, Kirmani S, Anand B. Virtual fracture clinic delivers British Orthopaedic Association compliance. Ann R Coll Surg Engl. 2017;99(1):51-4.

4. Waters S, Edmondston SJ, Yates PJ, Gucciardi DF. Identification of factors influencing patient satisfaction with orthopaedic outpatient clinic consultation: A qualitative study. Man Ther. 2016;25:48-55.

5. Wraighte PJ, Scammell BE. Principles of fracture healing. Surgery (Oxford). 2006;24(6):198-207.

6. Ferguson KB, McGlynn J, Jenkins P, Madeley NJ, Kumar CS, Rymaszewski L. Fifth metatarsal fractures - Is routine follow-up necessary? Injury. 2015;46(8):1664-8.

7. Jenkins PJ, Gilmour A, Murray O, Anthony I, Nugent MP, Ireland AJ, et al. The Glasgow Fracture Pathway: a virtual clinic. BJJ News. 2014(2):22-4.

8. Khan SA, Asokan A, Handford C, Logan P, Moores T. How useful are virtual fracture clinics?: a systematic review. Bone Jt Open. 2020;1(11):683-90.

9. Chan A-W, Tetzlaff JM, Altman DG, Laupacis A, Gøtzsche PC, Krleža-Jerić K, et al. SPIRIT 2013 Statement: Defining Standard Protocol Items for Clinical Trials. Annals of Internal Medicine. 2013;158(3):200-7.

10. Shaw M, Anderson T, Sinclair T, Hutchings O, Dearing C, Raffan F, et al. rpavirtual: Key lessons in healthcare organisational resilience in the time of COVID-19. Int J Health Plann Manage. 2022;37(3):1229-37.

11. Harris PA, Taylor R, Minor BL, Elliott V, Fernandez M, O'Neal L, et al. The REDCap consortium: Building an international community of software platform partners. Journal of Biomedical Informatics. 2019;95:103208.

12. Harris PA, Taylor R, Thielke R, Payne J, Gonzalez N, Conde JG. Research electronic data capture (REDCap)—A metadata-driven methodology and workflow process for providing translational research informatics support. Journal of Biomedical Informatics. 2009;42(2):377-81.

13. Novak CB, Williams MM, Conaty K. Evaluation of the Patient-Specific Functional Scale in hand fractures and dislocations. Hand (New York, NY). 2015;10(1):85-7.

For peer review only - http://bmjopen.bmj.com/site/about/guidelines.xhtml

14. Mannberg Bäckman S, Stråt S, Ahlström S, Brodin N. Validity and sensitivity to change of the Patient Specific Functional Scale used during rehabilitation following proximal humeral fracture. Disabil Rehabil. 2016;38(5):487-92.

15. Stratford P, Gill C, Westaway M, Binkley J. Assessing Disability and Change on Individual Patients: A Report of a Patient Specific Measure. Physiotherapy Canada. 1995;47(4):258-63.

16. Norman R, Mulhern B, Lancsar E, Lorgelly P, Ratcliffe J, Street D, et al. The Use of a Discrete Choice Experiment Including Both Duration and Dead for the Development of an EQ-5D-5L Value Set for Australia. Pharmacoeconomics. 2023;41(4):427-38.

17. Sjetne IS, Bjertnaes OA, Olsen RV, Iversen HH, Bukholm G. The Generic Short Patient Experiences Questionnaire (GS-PEQ): identification of core items from a survey in Norway. BMC Health Serv Res. 2011;11:88.

18. Bijur PE, Silver W, Gallagher EJ. Reliability of the visual analog scale for measurement of acute pain. Acad Emerg Med. 2001;8(12):1153-7.

19. Cleveland Clinic. Sample Size Estimation in Clinical Research https://riskcalc.org/samplesize/: Department of Quantitative Health Services; n.d. [

20. Navarro-Pujalte E, Gacto-Sanchez M, Montilla-Herrador J, Escolar-Reina P, Angeles Franco-Sierra M, Medina-Mirapeix F. Sensitivity to change of mobility measures in musculoskeletal conditions on lower extremities in outpatient rehabilitation settings. Disabil Rehabil. 2019;41(11):1268-74.

21. Abbott JH, Schmitt J. Minimum important differences for the patient-specific functional scale, 4 region-specific outcome measures, and the numeric pain rating scale. J Orthop Sports Phys Ther. 2014;44(8):560-4.

22. Pathak A, Wilson R, Sharma S, Pryymachenko Y, Ribeiro DC, Chua J, et al. Measurement Properties of the Patient-Specific Functional Scale and Its Current Uses: An Updated Systematic Review of 57 Studies Using COSMIN Guidelines. J Orthop Sports Phys Ther. 2022;52(5):262-75.

23. European Medicines Agency. Committee for Medicinal Products for Human Use (CHMP). Guideline on the Choice of the Non-inferiority Margin. London, United Kingdom: EMA; 2005.

For peer review only - http://bmjopen.bmj.com/site/about/guidelines.xhtml

24. Husereau D, Drummond M, Augustovski F, de Bekker-Grob E, Briggs AH, Carswell C, et al. Consolidated Health Economic Evaluation Reporting Standards 2022 (CHEERS 2022) Statement: Updated Reporting Guidance for Health Economic Evaluations. Value in Health. 2022;25(1):3-9.

25. Petrou S, Gray A. Economic evaluation alongside randomised controlled trials: design, conduct, analysis, and reporting. BMJ. 2011;342:d1548.

26. Braun V, Clarke V. Using thematic analysis in psychology. Qualitative Research in Psychology. 2006;3(2):77-101.

27. National Health and Medical Research Council. Safety monitoring and reporting in clinical trials involving therapeutic goods. Australian Research Council and Universities Australia: Commonwealth of Australia, Canberra; 2016.

28. National Health and Medical Research Council. Management of Data and Information in Research: A guide supporting the Australian Code for the Responsible Conduct of Research. Australian Research Council and Universities Australia: Commonwealth of Australia, Canberra; 2018.

FIGURE LEGEND

Figure 1: Trial design

PSFS: Patient Specific Functional Scale; EQ-5D-5L: EuroQol 5D 5L; Pain NRS: Pain Numerical Rating Scale; GS-PEQ: Generic Short Patient Experiences Questionnaire; AE & AE: Adverse events & serious adverse events; ED re-presentations: Emergency Department re-presentations.

For peer review only

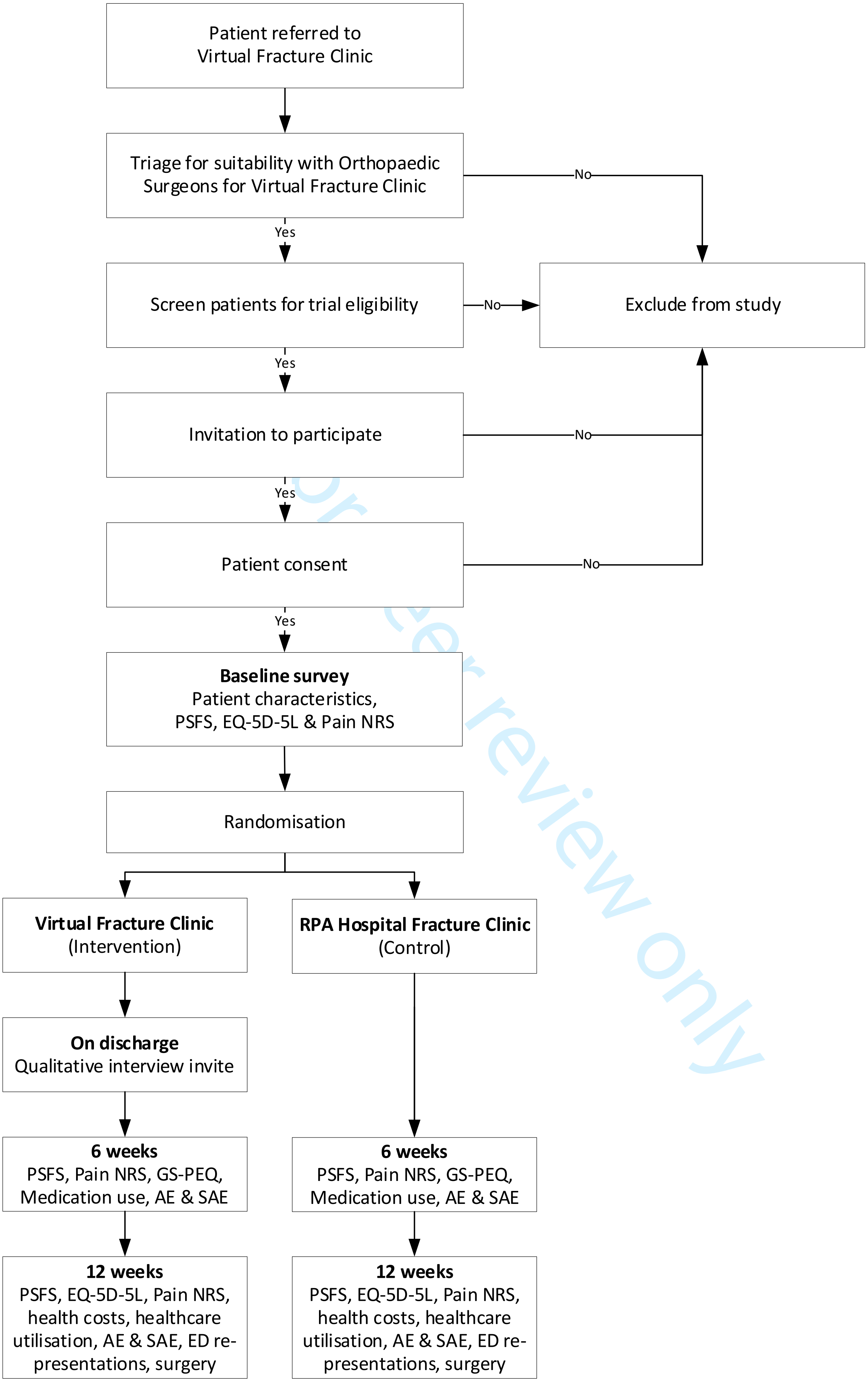

Figure 1: Trial design

For peer review only

PARTICIPANT INFORMATION SHEET

Do patients with simple fractures have similar functional outcomes when comparing a virtual against an in-person fracture clinic?

**Title**	Effects of virtual fracture clinic care compared with in-person fracture clinic care on physical function in people with simple fractures: a non-inferiority randomised trial.
**Short Title**	fractu**RE** **C**lin**I**c **T**ri**AL** (RECITAL)
**Protocol Number**	2023/ETH01038
**Project Sponsor**	RPA Virtual Hospital
**Coordinating Principal Investigator**	• Dr Adrian Traeger, Research Fellow, Institute for Musculoskeletal Health, The University of Sydney.
**Associate Investigator(s)**	• Mr Min Jiat Teng, Physiotherapist, RPA Virtual Hospital; Chief Investigator & PhD Candidate, Institute for Musculoskeletal Health.<br>• Ms Miranda Shaw, General Manager, RPA Virtual Hospital, SLHD.<br>• Dr Owen Hutchings, Clinical Director, RPA Virtual Hospital, SLHD.<br>• A/Prof Mark Horsley, Deputy Director Neurosciences, Bone & Joint, SLHD.<br>• Dr Jeffrey Petchell, Head of Orthopaedic Department & Director of Trauma, Royal Prince Alfred Hospital, SLHD.<br>• Prof Chris Maher, Director, Institute for Musculoskeletal Health, The University of Sydney.<br>• Dr Tessa Copp, Postdoctoral Research Fellow, Sydney Health Literacy Lab, The University of Sydney.<br>• Dr Kristen Pickles, Postdoctoral Research Fellow, Sydney Health Literacy Lab, The University of Sydney.<br>• Ms Rong Liu, Research Officer (Health Economics), RPA Virtual Hospital, SLHD.<br>• Ms Alison Drayton, Consumer Representative.<br>• Ms Isabella Khoudair, Physiotherapist, RPA Virtual Hospital, SLHD.<br>• Mr Ben Warnock, Physiotherapist, RPA Virtual Hospital, SLHD.
**Location**	• RPA Virtual Hospital<br>• RPA Fracture Clinic

For peer review only - http://bmjopen.bmj.com/site/about/guidelines.xhtml

1. Introduction

You are invited to take part in a research study that will compare two existing models of care for managing patients with simple fractures. The aim of the study is to understand whether virtual clinics are comparable to in-person clinics in terms of function, recovery and other outcomes.

The study is being conducted within Sydney Local Health District (SLHD) by Min Jiat Teng (Physiotherapist, SLHD) as part of the requirements for a Doctor of Philosophy degree under the supervision of Dr Adrian Traeger (Senior Research Fellow, Institute for Musculoskeletal Health).

This Participant Information Sheet (PIS) will tell you what is involved in the study and help you decide whether or not you wish to take part. Please read this information carefully. If there is anything you do not understand or would like further information about, please ask Min Jiat Teng on 0460 001 381 or min.teng@health.nsw.gov.au. Before you make a decision, please feel free to talk things over with a relative, a friend or your doctor.

2. Study Procedures

Your condition has been discussed with the orthopaedic doctors at RPA to ensure it can be managed at either the virtual clinic or in-person clinic.

If you agree to participate in this study, you will be asked to sign an e-consent form online via a link provided by the study team.

Once you give informed consent, you will be randomly assigned to receive your care with RPA in-person fracture clinic at Camperdown or via the virtual fracture clinic.

If you are assigned to our virtual clinic, you will receive care from a physiotherapist via video calls. There will be 3 scheduled appointments that take approximately 30 minutes each, and your follow-ups will usually be at 2 and 6 weeks after your injury. You will not need to travel to hospital for your appointments.

If assigned to our traditional in-person clinic, you will attend appointments here at the hospital on Missenden Road, Camperdown. You will be provided a check-in time with the clinic, and the staff will assess you and provide a follow-up plan based on the assessments.

We do not know if one of these two ways of following people up is better than the other.

You will receive a link via email or SMS to complete an online survey at 3 different time-points – when enrolled into the study, 6 weeks after that, and again in another 6 weeks. Each survey will take less than 5 minutes to complete. The surveys will ask about your experiences with the care you have received and aspects of your recovery. You will receive an email or SMS 2 days prior to each milestone, reminding you to complete your surveys. You will receive two reminders via email or SMS, followed by a phone call by a SLHD staff if you do not complete your surveys.

Your clinical records will be maintained either on paper or electronically at SLHD as per current processes. The surveys you complete will be stored online in the secure 'REDCap database', which is managed by SLHD. Your clinical and research data on REDCap will be de-identified, and can only be re-identified through a data linkage process using a unique ID code.*

Your medical records may be accessed by the clinicians listed in this study if they are relevant to this research. This may include your paper and electronic medical records from the hospital,

For peer review only - http://bmjopen.bmj.com/site/about/guidelines.xhtml

and/or the radiology scans and reports conducted out of the hospital (e.g. if your xrays were conducted at a private radiology centre).

Data from this study may be published in peer-reviewed medical journals, however you will not be personally identifiable.

If the study data will be used for future research purposes and/or shared with national and international collaborators, Ethics Approval will be required prior to accessing any non-identifiable data.

*Data linkage is a method of bringing together information, from different sources, but relating to the same individual.

Qualitative interview sub-study

Patients in the virtual clinic may be contacted after discharge inviting you to participate in a phone or online interview to explore your experiences with the virtual fracture clinic. The interview will take 30 to 60 minutes, and will be audio/video recorded. If you do not want a video recording, you will be able to turn off your camera in the Zoom meeting. Audio from the interview recording will be transcribed and will not contain any details that will identify you.

The interview will be conducted by researchers from The University of Sydney or SLHD who are not part of your treating team. The interview will ask you about your experiences, feelings and expectations of the virtual fracture clinic.

3. Risks

All medical procedures - whether for diagnosis or treatment, routine or experimental – involve some level of risk. The table below displays some of these risks and the respective mitigation strategy.

Possible Risk/Side Effect	When may this occur?	Mitigation strategy
Telehealth privacy risk	Small risk that patient data or information is intercepted electronically during videoconsults or emails.	The study will only use programs that are approved by SLHD for clinical records, videocalls, exercise programs and data management.
Clinical risk	Small risk that a condition may be missed or mis-diagnosed as the clinician is unable to physically assess patients via video consult.	Only patients who meet the strict clinical criteria are considered for this study. All cases will be screened with the orthopaedic surgeon to ensure clinical suitability to be managed either in person or by video consult.

If you wish to talk to someone outside the research team due to any distress caused to you by this study, you can contact:

- Executive Officer – Clinical Trials on 02 9515 8200.
- Research and Evaluation Manager, RPA Virtual Hospital on 02 9515 0248.
- Beyond Blue on 1300 224 636.
- Mental Health Line on 1800 011 511.

4. Benefits

For peer review only - http://bmjopen.bmj.com/site/about/guidelines.xhtml

Your participation in this study will also further medical knowledge and may improve treatment of virtual care for patients with simple injuries in the future.

5. Compensation for injuries or complications

If you suffer any injuries or complications as a result of this study, you should contact the study clinician as soon as possible, who will assist you in arranging appropriate medical treatment. If you are eligible for Medicare, you can receive any medical treatment required to treat the injury or complication, free of charge, as a public patient in any Australian public hospital.

In addition, you may have a right to take legal action to obtain compensation for any injuries or complications resulting from the study. Compensation may be available if your injury or complication is sufficiently serious and is caused by the services you received as part of this study, or by the negligence of one of the parties involved in the study (for example, the researcher, the hospital, or the treating doctor). You do not give up any legal rights to compensation by participating in this study.

6. Costs

Aside from giving up your time, there are no costs of participating in this study. You will receive a $50 eGift Card (WISH GiftCard) after completing all the surveys (at recruitment, 6 weeks and 12 weeks) to thank you for your time. You will receive an additional $50 eGift Card if you participate in the interview.

7. Voluntary Participation

Participation in this study is entirely voluntary. You do not have to take part. If you do take part, you can withdraw at any time without having to give a reason by contacting the study coordinator, Min Jiat Teng on 0460 001 381. Whatever your decision, please be assured that it will not affect your medical treatment or your relationship with the staff who are caring for you.

If you decide to withdraw from the study, we will not collect any more study-related information from you, although information already collected will be retained to ensure that the results of the research project can be measured properly and to comply with law. You should be aware that data collected up to the time you withdraw will form part of the research project results. If you do not want your data to be included, you must tell the researchers when you withdraw from the project. It will not be included in the study results, unless we have already analysed and published the results in aggregate form (you will not be personally identifiable).

8. Confidentiality

All the information collected from you for the study will be strictly confidential and will be stored on a secure research database (SLHD's REDCap server). This web-based software is managed and supported by the Clinical Research Centre and the SLHD Digital Health and Innovation (DH&I) department. This server is stable and is backed up daily in compliance with national, state and district privacy and confidentiality obligations. Only the investigators named on this research project or your treating clinicians will have access to it.

The data will be analysed by the researchers at RPA Virtual Hospital and The University of Sydney. All data included in journal publications and presentations will be de-identified*. The files will be retained for 15 years from the day the study is completed, after which they will be securely destroyed.

Any personally identifiable data such as your name, date of birth and e-consent form will be kept strictly confidential, separate from your survey data within REDCap. The data can only

For peer review only - http://bmjopen.bmj.com/site/about/guidelines.xhtml

be linked using a unique ID code. Only the named investigators and the clinicians providing care will have access to the data. The **rpa**virtual General Manager listed on this Participant Information Sheet will be the data custodian for this research.

*de-identified data means that you/your information will not be identifiable

9. Storage of Data

The SLHD software licence for REDCap (Research Electronic Data Capture) will be used for to manage the collection and storage of research data. REDCap is a secure, web-based, non-commercial, data management tool designed for research purposes. Data collected by REDCap is stored on servers in the SLHD data centre. Data is secured and backed-up to maintain your privacy and confidentiality in line with national, state and district standards.

10. Future use of Data

The data collected in this project may also be used in future research studies. The results of this study and de-identified data may be shared in the future with national and international collaborators. Any stored data that is used for related or future research will first be reviewed and approved by an appropriately constituted Ethics Committee.

11. Conflicts of Interest

The following investigators (AT, MS, OH, CM, TC, KP, JZ, IA, RL, AD) have no conflict on interests to declare. The SLHD clinicians (MJT, IK, BW, MH, JP) may deliver care to participants in either study groups as part of their usual clinical role provided at a public hospital. The SLHD clinicians will receive no financial or non-financial benefits for conducting this research, nor will RPA Virtual or RPA Hospitals receive any financial or other benefits.

Min Jiat Teng will be conducting this study in partial fulfillment of the requirements of a Doctor of Philosophy (Medicine and Health) degree under the supervision of Dr Adrian Traeger, Prof Christopher Maher, Dr Tessa Copp and Dr Kristen Pickles. This study has received funding from Sydney Research and the NHMRC 2022 MRFF Clinician Researchers - Nurses Midwives and Allied Health grant.

12. Further Information

When you have read this information, Min Jiat Teng will discuss it with you further and answer any questions you may have. If you would like to know more at any stage, please feel free to contact him on 0460 001 381 or min.teng@health.nsw.gov.au.

This information sheet is for you to keep.

13. Ethics Approval and Complaints

This study has been approved by the Ethics Review Committee (RPAH Zone) of the Sydney Local Health District. Any person with concerns or complaints about the conduct of this study should contact the Executive Officer on 02 9515 6766 and quote protocol number 2023/ETH01038.

For peer review only - http://bmjopen.bmj.com/site/about/guidelines.xhtml

Effects of virtual fracture clinic care compared with in-person fracture clinic care on physical function in people with simple fractures: a non-inferiority randomised trial.

Do patients with simple fractures have similar functional outcomes when comparing a virtual against an in-person fracture clinic?

PARTICIPANT CONSENT FORM

I, ____ *[full name]*

of ____ *[address]*

- I have read and understood the Participant Information Sheet on the above-named research study and have had the opportunity to discuss the study with the research team if required.
- I have been made aware of the procedures involved in the study, including any known or expected inconvenience, risk, discomfort or potential side effect and of their implications as far as they are currently known by the researchers.
- I understand that the interview discussion will be audio and/or video-recorded, and will then be transcribed and be kept in a manner in which I cannot be identified for analysis.
- I understand that my participation in this study will allow my clinicians and the study coordinator to have access to my medical record, as described in the Participant Information Sheet.
- I understand that my de-identified data may be used for future research.
- I would like to receive a copy of the study results when they become available.

  ❑ Yes ❑ No
- I understand that, during the course of this study, my medical records may be accessed by the research staff at RPA Virtual Hospital, by regulatory authorities or by the Ethics Committee approving the research in order to verify results and determine that the study is being carried out correctly.
- I understand that the SLHD software license for REDCap (Research Electronic Data Capture) will be used to manage the collection and storage of my research data.
- I have had an opportunity to ask questions and I am satisfied with the answers I have received.
- I freely choose to participate in this study and understand that I can withdraw at any time.
- I consent to the future use of any data I provide for research purposes. I understand that before they can use any data I provide, they must seek additional ethics approval.
- I understand that my participation and data will be kept strictly confidential and secure.
- I hereby agree to participate in this research study.
- I consent to the storage and use of my information collected from me for use, as described in the relevant section of the Participant Information Sheet, for:
  - This specific research project

For peer review only - http://bmjopen.bmj.com/site/about/guidelines.xhtml

- Other research that is closely related to this research project

My email address is ____

(a link to the survey will be send to this address)

Participant Name: ____

Participant Signature: ____

Date: ____

For peer review only

For peer review only - http://bmjopen.bmj.com/site/about/guidelines.xhtml

SPIRIT
STANDARD PROTOCOL ITEMS: RECOMMENDATIONS FOR INTERVENTIONAL TRIALS

SPIRIT 2013 Checklist: Recommended items to address in a clinical trial protocol and related documents*

Section/item	ItemNo	Description	Page & section of item in protocol
**Administrative information**			
Title	1	Descriptive title identifying the study design, population, interventions, and, if applicable, trial acronym	1 – Front Page
Trial registration	2a	Trial identifier and registry name. If not yet registered, name of intended registry	3 – Trial registration number<br>6 – Design
	2b	All items from the World Health Organization Trial Registration Data Set	Added as appendix
Protocol version	3	Date and version identifier	16 – Ethics and dissemination
Funding	4	Sources and types of financial, material, and other support	16 – Funding statement
Roles and responsibilities	5a	Names, affiliations, and roles of protocol contributors	1 – Front Page<br>16 – Author's contributions
	5b	Name and contact information for the trial sponsor	N/A
	5c	Role of study sponsor and funders, if any, in study design; collection, management, analysis, and interpretation of data; writing of the report; and the decision to submit the report for publication, including whether they will have ultimate authority over any of these activities	16 – Funding statement

For peer review only - http://bmjopen.bmj.com/site/about/guidelines.xhtml

	5d	Composition, roles, and responsibilities of the coordinating centre, steering committee, endpoint adjudication committee, data management team, and other individuals or groups overseeing the trial, if applicable (see Item 21a for data monitoring committee)	14 – Data monitoring<br>16 – Ethics and dissemination
**Introduction**			
Background and rationale	6a	Description of research question and justification for undertaking the trial, including summary of relevant studies (published and unpublished) examining benefits and harms for each intervention	3 – Introduction
	6b	Explanation for choice of comparators	3 – Introduction
Objectives	7	Specific objectives or hypotheses	3 – Introduction
Trial design	8	Description of trial design including type of trial (eg, parallel group, crossover, factorial, single group), allocation ratio, and framework (eg, superiority, equivalence, noninferiority, exploratory)	6 – Design
**Methods: Participants, interventions, and outcomes**			
Study setting	9	Description of study settings (eg, community clinic, academic hospital) and list of countries where data will be collected. Reference to where list of study sites can be obtained	6 – Setting
Eligibility criteria	10	Inclusion and exclusion criteria for participants. If applicable, eligibility criteria for study centres and individuals who will perform the interventions (eg, surgeons, psychotherapists)	6 – Eligibility criteria
Interventions	11a	Interventions for each group with sufficient detail to allow replication, including how and when they will be administered	8 – Interventions

For peer review only - http://bmjopen.bmj.com/site/about/guidelines.xhtml

	11b	Criteria for discontinuing or modifying allocated interventions for a given trial participant (eg, drug dose change in response to harms, participant request, or improving/worsening disease)	8 – Interventions
	11c	Strategies to improve adherence to intervention protocols, and any procedures for monitoring adherence (eg, drug tablet return, laboratory tests)	8 – Interventions
	11d	Relevant concomitant care and interventions that are permitted or prohibited during the trial	15 – Ancillary and post-trial care
Outcomes	12	Primary, secondary, and other outcomes, including the specific measurement variable (eg, systolic blood pressure), analysis metric (eg, change from baseline, final value, time to event), method of aggregation (eg, median, proportion), and time point for each outcome. Explanation of the clinical relevance of chosen efficacy and harm outcomes is strongly recommended	9 – Outcomes
Participant timeline	13	Time schedule of enrolment, interventions (including any run-ins and washouts), assessments, and visits for participants. A schematic diagram is highly recommended (see Figure)	Table 1 added as appendix
Sample size	14	Estimated number of participants needed to achieve study objectives and how it was determined, including clinical and statistical assumptions supporting any sample size calculations	10 – Sample size
Recruitment	15	Strategies for achieving adequate participant enrolment to reach target sample size	6 – Eligibility criteria

Methods: Assignment of interventions (for controlled trials)

Allocation:

For peer review only - http://bmjopen.bmj.com/site/about/guidelines.xhtml

Sequence generation	16a	Method of generating the allocation sequence (eg, computer-generated random numbers), and list of any factors for stratification. To reduce predictability of a random sequence, details of any planned restriction (eg, blocking) should be provided in a separate document that is unavailable to those who enrol participants or assign interventions	6 – Eligibility criteria
Allocation concealment mechanism	16b	Mechanism of implementing the allocation sequence (eg, central telephone; sequentially numbered, opaque, sealed envelopes), describing any steps to conceal the sequence until interventions are assigned	6 – Eligibility criteria
Implementation	16c	Who will generate the allocation sequence, who will enrol participants, and who will assign participants to interventions	6 – Eligibility criteria
Blinding (masking)	17a	Who will be blinded after assignment to interventions (eg, trial participants, care providers, outcome assessors, data analysts), and how	11 – Blinding
	17b	If blinded, circumstances under which unblinding is permissible, and procedure for revealing a participant's allocated intervention during the trial	N/A
**Methods: Data collection, management, and analysis**			
Data collection methods	18a	Plans for assessment and collection of outcome, baseline, and other trial data, including any related processes to promote data quality (eg, duplicate measurements, training of assessors) and a description of study instruments (eg, questionnaires, laboratory tests) along with their reliability and validity, if known. Reference to where data collection forms can be found, if not in the protocol	9 – Outcomes<br>11 – Data collection methods

For peer review only - http://bmjopen.bmj.com/site/about/guidelines.xhtml

	18b	Plans to promote participant retention and complete follow-up, including list of any outcome data to be collected for participants who discontinue or deviate from intervention protocols	12 – Statistical methods<br>12 – Secondary analysis<br>13 – Cost-effectiveness analysis
Data management	19	Plans for data entry, coding, security, and storage, including any related processes to promote data quality (eg, double data entry; range checks for data values). Reference to where details of data management procedures can be found, if not in the protocol	11 – Data management
Statistical methods	20a	Statistical methods for analysing primary and secondary outcomes. Reference to where other details of the statistical analysis plan can be found, if not in the protocol	12 – Statistical methods
	20b	Methods for any additional analyses (eg, subgroup and adjusted analyses)	13 – Cost-effectiveness analysis<br>13 – Qualitative interview analysis
	20c	Definition of analysis population relating to protocol non-adherence (eg, as randomised analysis), and any statistical methods to handle missing data (eg, multiple imputation)	12 – Statistical methods<br>12 – Secondary analysis<br>13 – Cost-effectiveness analysis
**Methods: Monitoring**			
Data monitoring	21a	Composition of data monitoring committee (DMC); summary of its role and reporting structure; statement of whether it is independent from the sponsor and competing interests; and reference to where further details about its charter can be found, if not in the protocol. Alternatively, an explanation of why a DMC is not needed	14 – Data monitoring
	21b	Description of any interim analyses and stopping guidelines, including who will have access to these interim results and make the final decision to terminate the trial	N/A

For peer review only - http://bmjopen.bmj.com/site/about/guidelines.xhtml

Harms	22	Plans for collecting, assessing, reporting, and managing solicited and spontaneously reported adverse events and other unintended effects of trial interventions or trial conduct	9 – Outcomes<br>14 – Harms
Auditing	23	Frequency and procedures for auditing trial conduct, if any, and whether the process will be independent from investigators and the sponsor	14 – Auditing
**Ethics and dissemination**			
Research ethics approval	24	Plans for seeking research ethics committee/institutional review board (REC/IRB) approval	16 – Ethics and dissemination
Protocol amendments	25	Plans for communicating important protocol modifications (eg, changes to eligibility criteria, outcomes, analyses) to relevant parties (eg, investigators, REC/IRBs, trial participants, trial registries, journals, regulators)	16 – Ethics and dissemination
Consent or assent	26a	Who will obtain informed consent or assent from potential trial participants or authorised surrogates, and how (see Item 32)	14 – Consent or assent
	26b	Additional consent provisions for collection and use of participant data and biological specimens in ancillary studies, if applicable	N/A
Confidentiality	27	How personal information about potential and enrolled participants will be collected, shared, and maintained in order to protect confidentiality before, during, and after the trial	15 – Access to data
Declaration of interests	28	Financial and other competing interests for principal investigators for the overall trial and each study site	17 – Competing interests statement

For peer review only - http://bmjopen.bmj.com/site/about/guidelines.xhtml

Access to data	29	Statement of who will have access to the final trial dataset, and disclosure of contractual agreements that limit such access for investigators	15 – Access to data
Ancillary and post-trial care	30	Provisions, if any, for ancillary and post-trial care, and for compensation to those who suffer harm from trial participation	15 – Ancillary and posts-trial care
Dissemination policy	31a	Plans for investigators and sponsor to communicate trial results to participants, healthcare professionals, the public, and other relevant groups (eg, via publication, reporting in results databases, or other data sharing arrangements), including any publication restrictions	15 – Dissemination policy
	31b	Authorship eligibility guidelines and any intended use of professional writers	15 – Dissemination policy
	31c	Plans, if any, for granting public access to the full protocol, participant-level dataset, and statistical code	15 – Access to data
**Appendices**			
Informed consent materials	32	Model consent form and other related documentation given to participants and authorised surrogates	Attached as Supplemental Material
Biological specimens	33	Plans for collection, laboratory evaluation, and storage of biological specimens for genetic or molecular analysis in the current trial and for future use in ancillary studies, if applicable	N/A

*It is strongly recommended that this checklist be read in conjunction with the SPIRIT 2013 Explanation & Elaboration for important clarification on the items. Amendments to the protocol should be tracked and dated. The SPIRIT checklist is copyrighted by the SPIRIT Group under the Creative Commons "Attribution-NonCommercial-NoDerivs 3.0 Unported" license.

For peer review only - http://bmjopen.bmj.com/site/about/guidelines.xhtml
